# Genetic Animal Models for Arrhythmogenic Cardiomyopathy

**DOI:** 10.3389/fphys.2020.00624

**Published:** 2020-06-24

**Authors:** Brenda Gerull, Andreas Brodehl

**Affiliations:** ^1^Comprehensive Heart Failure Center Wuerzburg, Department of Internal Medicine I, University Hospital Würzburg, Würzburg, Germany; ^2^Department of Cardiac Sciences, Libin Cardiovascular Institute of Alberta, University of Calgary, Calgary, AB, Canada; ^3^Erich and Hanna Klessmann Institute for Cardiovascular Research and Development, Heart and Diabetes Center NRW, University Hospitals of the Ruhr-University of Bochum, Bad Oeynhausen, Germany

**Keywords:** arrhythmogenic cardiomyopathy, desmosomes, animal models of human disease, sudden death, genetics, mouse, zebrafish

## Abstract

Arrhythmogenic cardiomyopathy has been clinically defined since the 1980s and causes right or biventricular cardiomyopathy associated with ventricular arrhythmia. Although it is a rare cardiac disease, it is responsible for a significant proportion of sudden cardiac deaths, especially in athletes. The majority of patients with arrhythmogenic cardiomyopathy carry one or more genetic variants in desmosomal genes. In the 1990s, several knockout mouse models of genes encoding for desmosomal proteins involved in cell–cell adhesion revealed for the first time embryonic lethality due to cardiac defects. Influenced by these initial discoveries in mice, arrhythmogenic cardiomyopathy received an increasing interest in human cardiovascular genetics, leading to the discovery of mutations initially in desmosomal genes and later on in more than 25 different genes. Of note, even in the clinic, routine genetic diagnostics are important for risk prediction of patients and their relatives with arrhythmogenic cardiomyopathy. Based on improvements in genetic animal engineering, different transgenic, knock-in, or cardiac-specific knockout animal models for desmosomal and nondesmosomal proteins have been generated, leading to important discoveries in this field. Here, we present an overview about the existing animal models of arrhythmogenic cardiomyopathy with a focus on the underlying pathomechanism and its importance for understanding of this disease. Prospectively, novel mechanistic insights gained from the whole animal, organ, tissue, cellular, and molecular levels will lead to the development of efficient personalized therapies for treatment of arrhythmogenic cardiomyopathy.

## Introduction

Arrhythmogenic cardiomyopathy (ACM) is a genetic cardiomyopathy characterized by ventricular arrhythmia often leading to sudden cardiac death in young people. Ventricular dysfunction often develops with progression of the disease leading to heart failure in some cases (Sen-Chowdhry and McKenna, 2010). ACM is frequently associated with fibro-fatty replacement of the myocardium (Slesnick et al., 2019). For a long time, the disease was referred to as arrhythmogenic right ventricular cardiomyopathy (ARVC) because the phenotype description was more focused on features of the right ventricle; however, increasing awareness of left ventricular and biventricular involvement led to the term “ACM” (Sen-Chowdhry et al., 2010). Because of the broad spectrum of ACM-related phenotypes sometimes overlapping with other cardiomyopathies, we follow in this review the recommendations of the Heart Rhythm Society (HRS) and focus on the genetic etiology of this disease (Protonotarios and Elliott, 2019; Towbin et al., 2019). The estimated prevalence of ACM is 1 in between 1000 and 5000 (Basso et al., 2009).

**FIGURE 1:**
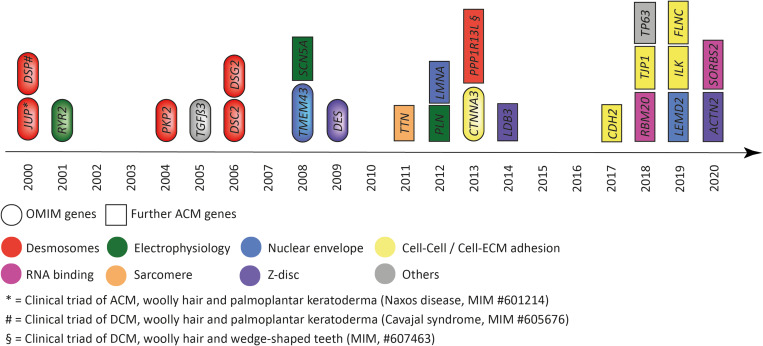
Genes associated with arrhythmogenic cardiomyopathy according to the year of discovery. Colors indicate the subcellular location and/or functional association. Eclipses indicate genes listed by the Online Mendelian Inheritance in the Man (OMIM) database. Squares indicate further genes associated with arrhythmogenic cardiomyopathy. *JUP* = plakoglobin, *DSP* = desmoplakin, *RYR2* = ryanodine receptor-2, *PKP2* = plakophilin-2, *TGFβ3* = transforming growth factor β3, *DSC2* = desmocollin-2, *DSG2* = desmoglein-2, *TMEM43* = transmembrane protein member-43, *SCN5A* = sodium voltage-gated channel subunit 5α, *DES* = desmin, *TTN* = titin, *PLN* = phospholamban, *LMNA* = lamin A/C, *CTNNA3* = αT-catenin, *PPP1R13L* = protein phosphatase 1 regulatory subunit 13 like, *LDB3* = LIM domain binding 3, *CDH2* = N-cadherin, *RBM20* = RNA binding motif protein 20, *TJP1* = tight junction protein-1, *TP63* = tumor protein-63, *LEMD2* = LEM-domain containing protein-2, *SORBS2* = sorbin and SH3 domain containing protein-2, *ILK* = integrin linked kinase, *FLNC* = filamin-C, *ACTN2* = actinin α2, *SORBS2* = sorbin, and SH3 = domain containing protein-2.

Discovery of genetic causes of ACM started with two recessive cardio-cutaneous syndromes named Naxos disease and Carvajal syndrome (McKoy et al., 2000; Norgett et al., 2000), which guided the discovery of autosomal dominant inherited nonsyndromic cardiomyopathy in the direction of disturbed cell–cell adhesion, in particular desmosomes (Figure 1). Pathogenic mutations in genes encoding desmosomal proteins account for about 50% of the genetic etiology (Klauke et al., 2010; Gandjbakhch et al., 2013). However, genetic mutations in additional genes expressed in different subcellular systems have been discovered by various genetic approaches, indicating extensive genetic heterogeneity (Figure 1).

Moreover, genetic findings brought up that the same gene, and sometimes even the same genetic variant, can be causative for a wide range of clinical features, indicating that disease entities are indistinct or clinically overlapping. For ACM, the clinical overlap occurs in particular with dilated cardiomyopathy (DCM) with which, sometimes, ventricular arrhythmia are the predominant phenotype and also with primary arrhythmia syndromes such as Brugada syndrome, in which a potential structural phenotype may not be overt at the time of presentation (Cerrone et al., 2014; Zegkos et al., 2020). In addition, it became more and more evident that not only the primary genetic cause, but also many other factors, such as environment, comorbidities, age, genetic background, and epigenetic factors, are contributing to disease onset, progression, and prognosis in patients with ACM (Bondue et al., 2018). Therefore, animal models provide a defined genetic background and are suitable to study the cause of disease as well as confounding variables under controlled and standardized conditions.

On the other hand, animal models also have several limitations when applied to mimic human cardiac disease, in particular the commonly used mouse and zebrafish models. For example, neither mice nor zebrafish develop cardiac fatty tissue, which is a hallmark for human ACM. Mice seem to be less prone to genetic defects affecting the heart, which often requires homozygous (recessive) models for mutations appearing heterozygous (dominant) in humans. Even more limited is the two-chamber heart of the zebrafish, which regenerates and may not be an ideal system to mimic specific aspects (e.g., right ventricular involvement) of ACM but has some advantages to study electrophysiology (Vornanen and Hassinen, 2016). Despite those limitations, animal models have contributed to a better understanding of the pathophysiology and molecular pathways leading to cardiomyopathy and the susceptibility to arrhythmia. Initially, often the animal model was used to analyze the pathological effect of a gene with consequences for the whole organism, the organ, and at the cellular and molecular levels. The discovery of cardiac phenotypes in animal models followed by a forward genetic approach led to identification of the genetic defect in the underlying human disease (Gerull et al., 2004; Grossmann et al., 2004). With the advances in genetic technology, often the disease gene in humans was first identified and subsequently modeled according to the mutation and proposed genetic mechanism (Heuser et al., 2006; Brodehl et al., 2019d). In this review, we evaluate the current state of animal models for ACM according to the proposed human disease–associated genes (Figure 1) and briefly discuss relevant mechanistic insights and limitations obtained from these studies. Some genes are more established than others or display a broad spectrum of phenotypes as part of an overlap syndrome. In particular, in the latter, we focus on models of mutations that have been associated with the ACM phenotype or representing a general mechanistic concept.

## Animal Models for ACM Associated With Mutations in Desmosomal Genes

**FIGURE 2:**
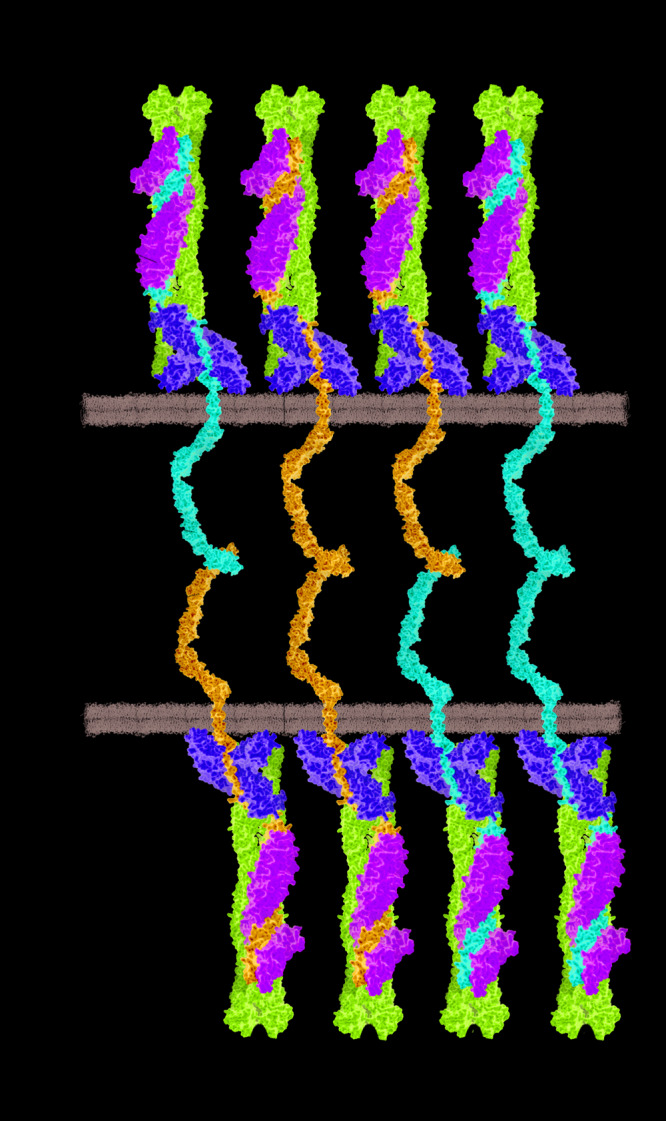
Schematic overview of cardiac desmosomes. DSC2 = desmocollin-2, DSG2 = desmoglein-2, PKP2 = plakophilin-2, PG = plakoglobin, DSP = desmoplakin.

Desmosomes are multiple protein complexes mediating cell–cell adhesion (Patel and Green, 2014). Especially in tissue exposed to mechanical forces, such as the skin and the heart during contraction, they have an important function for structural integrity. In addition, desmosomes are indirectly involved in the electrochemical coupling of cardiomyocytes (Cerrone and Delmar, 2014) and in signal transduction (Zhao et al., 2019). The molecular composition between cardiac and dermal desmosomes differs significantly (Goossens et al., 2007). For example, in the skin, all three plakophilins 1–3 are expressed, whereas cardiomyocytes express exclusively plakophilin-2^1^. Proteins from three different families build the desmosomes. In myocardial tissue, two desmosomal cadherins, desmocollin-2 and desmoglein-2 (encoded by *DSC2* and *DSG2*), mediate the intercellular, Ca^2+^-dependent adhesion (Harrison et al., 2016; Figure 2). The Ca^2+^ ion binding sites are localized in linker regions between the extracellular cadherin domains and are formed by aspartate and glutamate residues (Harrison et al., 2016). Both cadherins are type-I transmembrane proteins and carry several N-glycosylations and O-mannosylations in their extracellular domains (Harrison et al., 2016; Brodehl et al., 2019e; Debus et al., 2019). Their extracellular parts consist of five extracellular cadherin domains (ECD), mainly formed by β-sheets. Their first ECDs mediate this protein–protein interaction in *trans* position. Homophilic interactions between the desmosomal cadherins have relative high dissociation constants (K_*D*_ > 400 μM), whereas heterophilic interactions have higher affinities (K_*D*_ < 40 μM) (Harrison et al., 2016; Dieding et al., 2017). The intracellular domains of the desmosomal cadherins are bound in the heart by two proteins from the Armadillo family, which are called plakoglobin and plakophilin-2 (encoded by *JUP* and *PKP2*) (Chen et al., 2002; Figure 2). Central Armadillo domains consisting of several Armadillo repeats formed by a hairpin of two α-helices are the typical structural feature of these proteins (Choi et al., 2009; Kirchner et al., 2012). Plakoglobin and plakophilin-2 mediate the molecular interactions with the cytolinker protein desmoplakin (encoded by *DSP*) (Hofmann et al., 2000). Desmoplakin forms dimers and links the desmosomes to the intermediate filaments (IF), which consist in the heart mainly of the muscle-specific IF-protein desmin (encoded by *DES*) (Lapouge et al., 2006; Hatzfeld et al., 2017; Figure 2).

**FIGURE 3:**
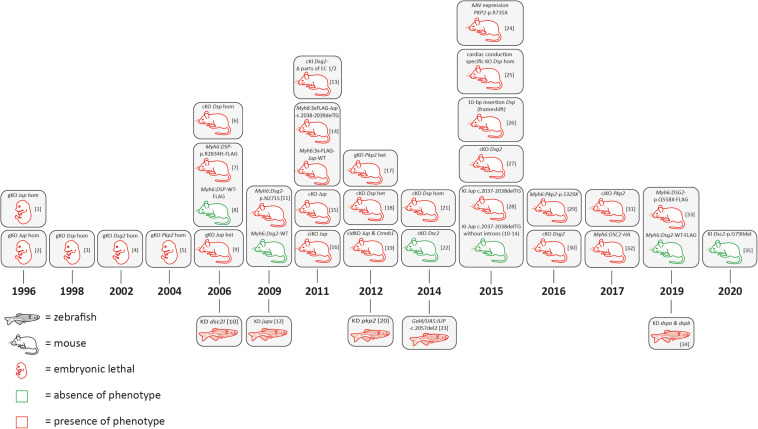
Schematic overview of animal models for desmosomal genes according to the year of publication. AAV = adeno associated virus; bp = base pair, cKO = cardiac specific knockout; ciKO = cardiac specific, inducible knockout; cidKO = cardiac specific, inducible double knockout; gKO = global knockout; het = heterozygous; hom = homozygous; KD = knockdown; KI = knock-in. [1] (Ruiz et al., 1996); [2] (Bierkamp et al., 1996); [3] (Gallicano et al., 1998); [4] (Eshkind et al., 2002); [5] (Grossmann et al., 2004); [6] (Garcia-Gras et al., 2006; Cheedipudi et al., 2019); [7] (Yang et al., 2006); [8] (Kirchhof et al., 2006; Fabritz et al., 2011); [9] (Heuser et al., 2006); [10] (Pilichou et al., 2009; Rizzo et al., 2012); [11] (Martin et al., 2009); [12] (Krusche et al., 2011; Kant et al., 2012; Buck et al., 2018); [13] (Lombardi et al., 2011); [14] (Li et al., 2011); [15] (Li et al., 2011); [16] (Cerrone et al., 2012; Leo-Macias et al., 2015); [17] (Gomes et al., 2012); [18] (Swope et al., 2012); [19] (Moriarty et al., 2012); [20] (Lyon et al., 2014); [21] (Rimpler, 2014); [22] (Asimaki et al., 2014); [23] (Cruz et al., 2015); [24] (Mezzano et al., 2016); [25] (Herbert Pratt et al., 2015); [26] (Kant et al., 2015); [27] (Zhang et al., 2015); [28] (Moncayo-Arlandi et al., 2016); [29] (Chelko et al., 2016); [30] (Cerrone et al., 2017); [31] (Brodehl et al., 2017); [32] (Calore et al., 2019); [33] (Giuliodori et al., 2018); [34] (Hamada et al., 2020).

In the 1990s, the relevance of desmosomal genes for ACM was recognized by the attempt to generate global knockout mice. Independently, the groups of Kemler and Birchmeier demonstrated that global *Jup* deficiency causes embryonic lethality in mice due to severe cardiac defects (Bierkamp et al., 1996; Ruiz et al., 1996; Figure 3 and Supplementary Tables 1, 2). The number of cardiac desmosomes was significantly reduced in the *Jup*-deficient embryos. Correspondingly, it was also shown that the global knockout of *Dsp*, *Dsg2*, and *Pkp2* causes embryonic lethality in mice due to severe heart defects (Gallicano et al., 1998; Eshkind et al., 2002; Grossmann et al., 2004). Of note, these initial findings stimulated the human cardiovascular genetic field significantly. In consequence, genetic analyses of human-related ACM patients revealed pathogenic mutations in the desmosomal genes *JUP* (McKoy et al., 2000), *DSP* (Norgett et al., 2000), *PKP2* (Gerull et al., 2004), *DSG2* (Awad et al., 2006; Pilichou et al., 2006), and *DSC2* (Heuser et al., 2006; Syrris et al., 2006; Figure 1). Patients carrying a homozygous 2-bp *JUP* deletion presented, in addition to ACM, also wooly hair and palmoplantar keratoderma. Because these patients were from the Greek island Naxos, this clinical triad is also known as Naxos disease (MIM, #601214) (Protonotarios and Tsatsopoulou, 2006). Patients with pathogenic mutations in the *DSP* gene frequently presented with a comparable triad of clinical features consisting of DCM, wooly hair, and palmoplantar keratoderma, which is known as Carvajal syndrome (MIM, #605676) (Protonotarios and Tsatsopoulou, 2004). An additional skeletal muscle myopathy can contribute to the phenotype of patients with mutations in *DES* or *FLNC* (Klauke et al., 2010; Verdonschot et al., 2020).

In conclusion, initial findings in mice and humans led to the hypothesis that ACM is mainly a disease of cardiac desmosomes supported by the fact that about 50% of ACM patients carry one or multiple mutations in desmosomal genes (Xu et al., 2010). Despite the stimulating effects for human cardiovascular genetics, embryonic lethality associated with global desmosomal gene deficiency limited the molecular analyses of the underlying pathomechanisms involved in ACM. Cellular models, including cardiomyocytes derived from human-induced pluripotent stem cells (hiPSC), might be an alternative approach for *in vitro* analyses (for a review about hiPSC ACM models, see Brodehl et al. (2019a). In addition, different transgenic, knock-in (KI), and conditional cardiac-specific mouse models for the desmosomal genes have been developed to overcome embryonic lethality (Figure 3 and Supplementary Tables 1, 2). Different animal studies revealed that the number of cardiac desmosomes is decreased. In addition, the ultrastructure of the cardiac desmosomes is also significantly changed (Heuser et al., 2006). Both effects might contribute to a widening of the intercalated disc. In consequence, the electrical coupling of adhesive cardiomyocytes through gap junctions might be indirectly affected, which can explain an increased risk for ventricular arrhythmia in ACM patients. This hypothesis is in good agreement with studies demonstrating a remarkable mislocalization of connexin-43 at the intercalated disc, which is the main cardiac gap junction protein (Gomes et al., 2012). More recently, molecular and functional crosstalk between plakophilin-2 and the voltage-gated sodium channel complex was discovered, indicating that the sodium channel current is decreased (Agullo-Pascual et al., 2013, 2014; Cerrone and Delmar, 2014).

In summary, the electrophysiological pathomechanisms caused by defects in desmosomal proteins are complex and still a matter of debate. Presumably, genetic, biochemical and electrophysiological changes contribute in combination to the complex disease phenotype of ACM.

### Plakoglobin

In general, there is still an ongoing debate about if a loss-of-function (LOF) pathomechanism or a toxic effect of mutant plakoglobin is involved in ACM. More than 50 different rare human *JUP* variants are currently listed in the Human Gene Mutation Database (HGMD^2^) (Krawczak et al., 2000). For several of the known *JUP* missense variants, the significance is unknown, or they are even classified as (likely) benign. However, homozygous truncating mutations in *JUP* are almost certainly pathogenic (McKoy et al., 2000). To overcome the embryonic lethality of global *Jup* deficiency in mice, additional mice and zebrafish models have been generated (Figure 3 and Supplementary Tables 1, 2). Kirchhof and colleagues demonstrated that even heterozygous global *Jup* knockout mice developed right ventricular dilation, ventricular arrhythmia, and decreased right ventricular function without developing structural defects (Kirchhof et al., 2006; Fabritz et al., 2011). Interestingly, exercise strengthened and accelerated cardiac disease manifestation. For endurance training, the authors used a swimming approach over 8 weeks with an increasing time period (5–90 min) and observed an increased right ventricular dilation and dysfunction after 6 months (Kirchhof et al., 2006). In good agreement, knockdown via injection of morpholino oligonucleotides induced a cardiac phenotype consisting of cardiac edema, decreased heart size, and blood reflux between the two cardiac chambers in zebrafish (*Danio rerio*) (Martin et al., 2009). In addition, cardiac-specific or inducible cardiac-specific plakoglobin-deficient mice developed severe cardiac fibrosis in combination with cardiac dysfunction, ventricular arrhythmia, and dilation (Li et al., 2011; Li et al., 2011). These findings support an LOF pathomechanism for *JUP* mutations involved in ACM. In contrast, transgenic mice with a cardiac-specific overexpression of mutant or even wild-type plakoglobin under the control of the cardiac-specific *Myh6* promotor caused an increased mortality in mice (Lombardi et al., 2011). Likewise, transgenic zebrafish with an overexpression of mutant plakoglobin developed a comparable phenotype (Asimaki et al., 2014). In 2015, the Chen’s group generated two remarkably different KI *Jup* mice, carrying a 2-bp deletion in exon 11 leading to a frame shift (*Jup*-c.2037-2038del) (Zhang et al., 2015). Regular knock-in mice died shortly after birth and showed decreased myocardial expression of mutant plakoglobin in comparison to the wild-type control mice, indicating that nonsense-mediated mRNA-decay (NMD) might be involved. Exon–exon junction complexes are involved in NMD (Dyle et al., 2020). In the second KI mice, carrying exactly the same 2-bp deletion in *Jup*, the introns 10–14 were deleted to block genetically NMD. Of note, this procedure rescued the expression of mutant and truncated plakoglobin and prevented the development of an ACM phenotype in these mice, supporting a clear LOF pathomechanism (Zhang et al., 2015). In conclusion, different animal models demonstrate that a balanced homeostasis of plakoglobin is necessary for normal cardiac function. There are several arguments that properties of pathogenic mutations in *JUP* might be caused by LOF. However, it cannot be completely excluded that *JUP* mutations have, in addition, toxic effects.

### Desmoplakin

According to the HGMD, currently, nearly 200 different human cardiomyopathy-causing *DSP* mutations are known. In 5%–10% of all ACM patients, a pathogenic *DSP* mutation can be identified (Gerull et al., 2019). Homozygous and heterozygous truncating mutations spread over the total *DSP* length are common. In contrast to plakoglobin, the cardiac-specific overexpression of the wild-type desmoplakin is not toxic (Yang et al., 2006; Figure 3). However, transgenic mice with cardiac-specific overexpression of mutant desmoplakin (*Myh6:DSP*-p.R2834H-FLAG) developed cardiac fibrosis and apoptosis, leading to ventricular dilation in combination with cardiac dysfunction and ultrastructural changes of the intercalated disc (Yang et al., 2006). On the other hand, an LOF pathomechanism for *DSP* mutations is supported by generation and characterization of cardiac-specific *Dsp* deficient mice, which causes the homozygous status embryonic lethality (Garcia-Gras et al., 2006). Heterozygous, cardiac-specific *Dsp* knockout mice developed fibro-fatty replacement of the myocardium and increased cardiomyocyte death, leading to cardiac dysfunction and ventricular arrhythmia supporting haploinsufficiency as the underlying pathomechanism (Garcia-Gras et al., 2006). Interestingly, genes encoding inhibitors of the WNT pathway and inflammatory proteins were differently expressed in these mice (Cheedipudi et al., 2019). Knockdown of *dspa* and *dspb* in zebrafish using morpholino injections showed, in good agreement, altered WNT, TGFβ, and Hippo/YAP-TAZ signaling (Giuliodori et al., 2018). Downregulation of connexins, which are the protein building blocks of the gap junctions, was described in heterozygous and homozygous cardiac specific *Dsp*-deficient mice (Gomes et al., 2012; Lyon et al., 2014). In 2015, Mezzano et al. generated a mouse model with a cardiac conduction system–specific *Dsp* ablation, revealing the impact of desmoplakin for the cardiac pacemaker function (Mezzano et al., 2016). As human *DSP* mutations also cause Carvajal syndrome in addition to isolated ACM, a spontaneous homozygous *Dsp*^*rul*^ mouse model deserves attention. *Dsp*^*rul*^ mice carry a spontaneous 10-bp insertion, leading to a frame shift and, consequently, to a premature termination codon in the C-terminal part of desmoplakin. These mice developed abnormal coat and epidermal blistering and presented, in addition, electrophysiological alterations and cardiac fibrosis (Herbert Pratt et al., 2015). In summary, the majority of animal models demonstrate an LOF mechanism for *DSP*, which is, moreover, supported in humans by the relatively high amount (5%–10%) of pathogenic truncating *DSP* mutations associated with an ACM phenotype.

### Plakophilin-2

Mutations in *PKP2* are common in ACM, and more than 250 different pathogenic mutations spread over the whole sequence are listed currently in the HGMD. The majority of *PKP2* mutations are truncating mutations although also pathogenic missense mutations are known (Kirchner et al., 2012). Presumably, haploinsufficiency is the major pathomechanism caused by *PKP2* mutations (Kirchner et al., 2012; Rasmussen et al., 2014). However, even a homozygous deletion of *PKP2* was described in two siblings of a consanguineous family. Both siblings presented severe left-ventricular noncompaction cardiomyopathy (LVNC) instead of ACM (Ramond et al., 2017). Several different animal models support the concept of *PKP2* haploinsufficiency (Figure 3). Knockdown of *pkp2* in zebrafish caused cardiac edema, incomplete heart looping, and a decreased heart rate. The structure of the cardiac desmosomes was, moreover, altered in these zebrafish (Moriarty et al., 2012). Because homozygous global *Pkp2* deficiency is embryonically lethal in mice (Grossmann et al., 2004), heterozygous *Pkp2*-deficient mice were used for functional analyses (Cerrone et al., 2012; Leo-Macias et al., 2015). Although no histological phenotype was present in the heterozygous animals, they developed electrophysiological abnormalities and ultrastructural defects (Cerrone et al., 2012; Leo-Macias et al., 2015). However, two overexpression models of truncated plakophilin-2 might indicate a toxic effect in mice (Cruz et al., 2015; Moncayo-Arlandi et al., 2016). Cruz et al. used adeno-associated viruses (AAVs) to express mutant truncated plakophilin-2 (p.R375X). In combination with exercise, this overexpression led to right-ventricular dysfunction. Those findings are similar to a transgenic mouse model with a cardiac-specific overexpression of Pkp2-p.S329X. Although, no fibro-fatty replacement was present in these transgenic mice, electrophysiological abnormalities and ultrastructural defects were present. In addition, a decreased expression of the other desmosomal proteins and remodeling of connexin-43 was found (Moncayo-Arlandi et al., 2016). Recently, Cerrone et al. developed a cardiac-specific inducible *Pkp2* mice line presenting decreased ventricular function, severe cardiac fibrosis, and arrhythmia. Interestingly, remodeling of genes involved in Ca^2+^ handling was found using this mouse model (Cerrone et al., 2017). Overall, it may be summarized that there is high evidence that *Pkp2* haploinsufficiency is involved in the ACM-associated pathomechanism.

### Desmoglein-2 and Desmocollin-2

Heterozygous and homozygous pathogenic mutations in *DSG2* and *DSC2*, encoding the two cardiac desmosomal cadherins, have been identified in human ACM patients responsible for about 5% of cases (Gerull et al., 2019; Brodehl et al., 2020). Using morpholino oligonucleotide injections, Heuser et al. knocked down *dsc2l* in zebrafish larvae causing cardiac edema, decreased fractional shortening, and altered desmosomal structure (Heuser et al., 2006). In contrast, homozygous conditional *Dsc2*-deficient mice were, under normal housing conditions, vital and did not develop cardiac dysfunction or show an increased mortality (Rimpler, 2014). Interestingly, homozygous knock-in mice carrying *Dsc2*-p.G790del did not develop a cardiac phenotype (Hamada et al., 2020). However, cardiac-specific overexpression of wild-type desmocollin-2 causes severe biventricular cardiomyopathy, including severe fibrotic scar formation, cardiac necrosis, calcification, and aseptic inflammation (Brodehl et al., 2017). Nevertheless, this mouse model cannot be directly transferred to the human condition of *DSC2* mutation carriers. Therefore, it is currently unknown if LOF or a pathogenic dosage effect of mutant desmocollin-2 contributes to ACM development.

The situation for desmoglein-2 is even more complex. Two transgenic mouse models with a cardiac-specific overexpression of Dsg2-p.N271S and DSG2-p.Q554X have been generated. The control mice with a cardiac-specific overexpression of the wild-type *DSG2* did not show any structural or functional abnormalities (Pilichou et al., 2009; Calore et al., 2019). In contrast, transgenic *Myh6:Dsg2*-p.N271S mice developed arrhythmia and ultrastructural defects of the intercalated disc (Pilichou et al., 2006). Correspondingly, *Myh6:*DSG2-p.Q554X mice developed cardiac fibrosis and had a reduced number of cardiac desmosomes. Interestingly, these mice had an increased expression of miRNAs signatures, which might be involved in the pathogenesis (Calore et al., 2019). The group of Krusche and Leube developed a mutant *Dsg2* mouse line missing parts of the first extracellular domains 1–2, mediating the homophilic and heterophilic intercellular protein–protein interactions. These mice developed cardiac fibrosis, necrosis, and calcification and showed an increased expression of cardiac stress markers. Ventricular arrhythmia and ultrastructural defects of the cardiac desmosomes were present in these mice (Krusche et al., 2011; Kant et al., 2012; Buck et al., 2018). Cardiac-specific *Dsg2* knockout mice were independently developed by two groups (Kant et al., 2015; Chelko et al., 2016). These mice present typical ACM features, leading to decreased cardiac function and arrhythmia. Of note, Chelko et al. demonstrated using *Dsg2*-deficient mice for which pharmacological inhibition of glycogen synthase kinase 3β (GSK3β) is able to improve cardiac function, indicating a putative therapeutic strategy for ACM as previously found in a transgenic zebrafish model (Asimaki et al., 2014; Chelko et al., 2016). However, whether inhibition of GSK3β serves as a therapeutic strategy in humans needs to be tested in future clinical trials. In addition, the molecular mechanism of GSK3ß inhibition in the context of ACM is currently unknown.

### iASPP

Recently, Notari et al. demonstrated that the inhibitor of apoptosis-stimulating protein of p53 (iASPP) is a cardiac desmosomal protein by binding to desmoplakin and desmin (Notari et al., 2015). However, iASPP is also known as NFκB-interacting protein-1 (NKIP1) and has a widespread expression in different organs and cell types^3^. Of note, a different group identified a homozygous nonsense mutation in *PPP1R13L*, which encodes iASPP, in a consanguineous family by whole exome sequencing. The infant patients showed severe DCM, cardiac fibro-fatty replacement, tachycardia, wooly hair, and wedge-shaped teeth but no palmoplantar keratoderma (Falik-Zaccai et al., 2017). A spontaneous deletion affecting a splice site in *Ppp1r13l* in a mouse model caused abnormal coat, growth retardation, increased prenatal mortality, and biventricular cardiomyopathy with massive cardiac fibrosis (Herron et al., 2005). More recently, an inducible *Ppp1r13l* knockout mouse model was generated, which presented an ACM phenotype consisting of right ventricular dilation, ventricular tachycardia, and ultrastructural changes of the cardiac desmosomes (Notari et al., 2015). In addition, a homozygous 7-bp duplication in *PPP1R13L* leading to a frame shift and a premature stop codon is responsible in Poll Hereford cattle for correspondent cardiac and coat abnormalities, leading to prenatal death (Simpson et al., 2009).

## Animal Models for ACM Associated With Mutations in Nondesmosomal Genes

**FIGURE 4:**
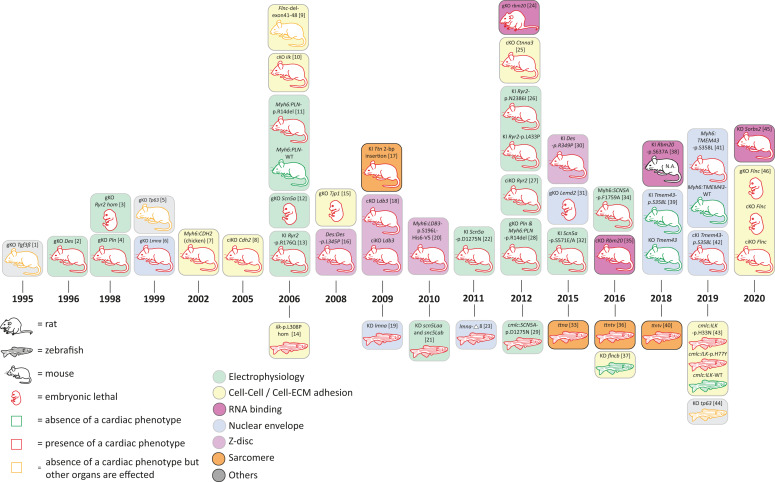
Schematic overview of animal models for non-desmosomal genes according to the year of publication. bp = base pair, KD = knock-down; cKO = cardiac specific knock-out; ciKO = cardiac specific, inducible knock-out; gKO = global knock-out; hom = homozygous; KI = knock-in. N.A. = not assessed. [1] (Kaartinen et al., 1995); [2] (Li et al., 1996; Capetanaki et al., 1997); [3] (Takeshima et al., 1998); [4] (Chu et al., 1998); [5] (Mills et al., 1999); [6] (Sullivan et al., 1999; Nikolova et al., 2004); [7] (Ferreira-Cornwell et al., 2002); [8] (Kostetskii et al., 2005; Li et al., 2005); [9] (Dalkilic et al., 2006); [10] (White et al., 2006; Quang et al., 2015); [11] (Haghighi et al., 2006); [12] (Liu et al., 2006); [13] (Kannankeril et al., 2006); [14] (Bendig et al., 2006; Pott et al., 2018); [15] (Katsuno et al., 2008); [16] (Kostareva et al., 2008); [17] (Gramlich et al., 2009); [18] (Zheng et al., 2009); [19] (Vogel et al., 2009); [20] (Li et al., 2010); [21] (Chopra et al., 2010); [22] (Watanabe et al., 2011); [23] (Koshimizu et al., 2011); [24] (Guo et al., 2012); [25] (Li et al., 2012); [26] (Shan et al., 2012); [27] (Bround et al., 2012); [28] (Haghighi et al., 2012) [29] (Huttner et al., 2013); [30] (Clemen et al., 2015; Stockigt et al., 2020); [31] (Tapia et al., 2015); [32] (Glynn et al., 2015); [33] (Zou et al., 2015); [34] (Wan et al., 2016); [35] (Khan et al., 2016; van den Hoogenhof et al., 2018); [36] (Shih et al., 2016); [37] (Begay et al., 2018); [38] (Murayama et al., 2018); [39] (Stroud et al., 2018); [40] (Huttner et al., 2018); [41] (Padron-Barthe et al., 2019); [42] (Zheng et al., 2019); [43] (Brodehl et al., 2019d); [44] (Santos-Pereira et al., 2019); [45] (Ding et al., 2019); [46] (Zhou et al., 2020).

In addition to mutations in desmosomal genes, several rare mutations in nondesmosomal genes have been identified in human ACM patients. Those ACM-associated genes encode proteins involved in cardiac electrophysiology (encoded by *RYR2*, *SCN5A*, *PLN*), Z-band proteins (encoded by *DES*, *LDB3*, *ACTN2*), nuclear envelope proteins (encoded by *TMEM43*, *LMNA*, *LEMD2*), or proteins involved in cell–cell or cell to extracellular matrix (ECM) adhesion (encoded by *CTNNA3*, *CDH2, TJP1*, *ILK*, *FLNC*) (Figures 1, 4 and Supplementary Tables 1, 2).

### Animal Models for ACM Genes Involved in Cardiac Electrophysiology

The human ACM phenotype is associated with severe ventricular arrhythmia, often leading to sudden cardiac death at first presentation. Therefore, mutations in proteins regulating ion channels and calcium signaling have been proposed as part of overlapping phenotypes of ACM with primary arrhythmia syndromes and DCM. The three main representative genes are *RYR2*, *SCN5A*, and *PLN*.

#### Ryanodine Receptor 2

The cardiac ryanodine receptor 2 gene (*RYR2*) has been proposed as the first nonsyndromic autosomal dominant inherited disease gene for ACM (MIM #600996; Tiso et al., 2001). Although the separate entity ACM has been subsequently questioned as mutations in *RYR2* typically lead to exercise or emotion-induced ventricular tachycardia without overt cardiomyopathy, referred to as catecholaminergic ventricular tachycardia (CPVT). Interestingly, three of the four originally described human mutations have been modeled in mice. The Ryr2*-p.*R176Q^+/–^ knock-in mouse (Kannankeril et al., 2006) mimics the human p.R176Q mutation and shows decreased right ventricular end-diastolic volumes without histological changes and ventricular tachycardia after beta-adrenergic stimulation. Two other human mutations related to ACM have been analyzed in mice (Shan et al., 2012). However, both Ryr2-p.L433P^+/–^ and Ryr2-p.N2386I^+/–^ knock-in mice demonstrated induced atrial fibrillation and leaky calcium channels in the sarcoplasmic reticulum of atrial myocytes but no structural or functional changes of cardiomyopathy. In contrast to mouse models of *Ryr2* representing gain-of-function mutations, the LOF of *Ryr2* in the global knockout mouse appears to be embryonically lethal at E10 with developmental defects, such as abnormalities in the heart tube morphology, large vacuolate sarcoplasmic reticulum, and structurally abnormal mitochondria (Takeshima et al., 1998). Moreover, an induced acute loss of half of the Ryr2 protein in the adult mouse hearts (Bround et al., 2012) leads to severe cardiac dysfunction, decreased heart rate, and ventricular arrhythmia, indicating that LOF not only causes fatal arrhythmia, but also has a role in heart rate control and cardiac remodeling contributing to heart failure.

#### Sodium Voltage-Gated Channel α Subunit 5

Mutations in *SCN5A* encoding the voltage-gated sodium channel subunit alpha Nav1.5 can cause cardiomyopathies and channelopathies. Functionally, there is a link to the desmosome as loss of *PKP2* expression alters the amplitude and kinetics of the sodium current (Sato et al., 2011; Cerrone et al., 2012). The first description of an ACM-like phenotype with an *SCN5A* variant came from a clinical study in which a coved-type ST-segment was provoked in ACM patients by a sodium channel blocker (Peters, 2008). Subsequently, a larger study found in almost 2% of ACM patients rare *SCN5A* variants and could show for *SCN5A*-p.R1898H reduced peak sodium current and abundance of NaV1.5 in clusters at the intercalated disc (Te Riele et al., 2017). Classically, gain-of-function *SCN5A* mutations cause Long QT syndrome, whereas LOF mutations are responsible for Brugada syndrome. However, both types of mutations may cause structural changes, usually in the form of DCM (Wilde and Amin, 2018). So far, the question remains whether the cardiomyopathy is a direct consequence of the mutation because Nav1.5 interacts with the proteins of the cytoskeleton and intercalated discs or may be secondary due to conduction defects caused by LOF effects. For example, mice with reduced 90% *Scn5a* expression develop conduction defects and severe biventricular cardiomyopathy (Hesse et al., 2007), also seen in a mouse and zebrafish model mimicking the human p.D1275N mutation. Mice homozygous (DN/DN) for the humanized mutation show conduction slowing and progressive cardiac dysfunction but no fibrosis (Watanabe et al., 2011). The transgenic zebrafish expressing the same human p.D1275N mutation exhibit bradycardia, conduction-system abnormalities, and premature death but no impaired cardiac function (Huttner et al., 2013).

Reduced *Scn5a* of about 50% as seen in heterozygous knockout hearts showed also impaired atrioventricular conduction and delayed intraventricular conduction, increased ventricular refractoriness, and ventricular tachycardia with characteristics of reentrant excitation. Homozygous mice were embryonically lethal with severe defects in ventricular morphology (Papadatos et al., 2002). Likewise, knockdown of cardiac sodium channels in zebrafish compromised both early chamber formation and normal patterned growth of the ventricle, suggesting that cardiac sodium channels in zebrafish affect heart development via a nonelectrophysiological mechanism (Chopra et al., 2010).

Another mouse model that modeled the human p.F1759A mutation showed that incomplete NaV1.5 channel inactivation is able to drive structural alterations, including atrial and ventricular enlargement, myofibrillar disarray, fibrosis and mitochondrial injury, and electrophysiological dysfunctions (Wan et al., 2016). Moreover, a mouse model is focusing on the phosphorylation residue S571 in two *Scn5a* knock-in mouse models (p.S571E and p.S571A). Interestingly, the authors could demonstrate a binary molecular switch for CaMKII-dependent activation of *I*_*Na,L*_ without affecting channel properties related to cardiac function (Glynn et al., 2015).

So far, none of the genetic variants found in typical ACM patients (Te Riele et al., 2017) have been analyzed in an animal model to specifically look at changes of the right ventricle; however, it is likely that LOF mutations are associated with cardiac dysfunction of the left or both ventricles even if whether a gain-of-function mechanism due to an interaction with desmosomal proteins exists remains unclear.

#### Phospholamban

Disturbed calcium homeostasis has been implicated as a mechanism for arrhythmogenesis as well as disturbed cardiac contractility. Phosholamban (encoded by *PLN*) regulates calcium handling by reversibly inhibiting the activity of the sarcoplasmic reticulum calcium ATPase 2 (SERCA2). This is based on the phosphorylation status through beta-adrenergic activation of protein kinase A (PKA). Although many human mutations have been described to cause DCM, an in-frame 3-bp deletion mutation leading to removal of R14 in phospholamban has been reported to cause both DCM and ACM, indicating the clinical overlap of both cardiomyopathies in particular for this mutation (van der Zwaag et al., 2012; Fish et al., 2016). *PLN*-p.R14del appears to be associated with high penetrance and severe disease. Two mouse models have been generated to mimic human disease. The transgenic mouse overexpressing the human PLN-p.R14del in the heart revealed ventricular dilation, myocyte disarray, and myocardial fibrosis. Mechanistically, it has been proposed that the dominant effect of the mutation leads to a nonreversible chronic suppression of SERCA activity based on its lack of PKA-mediated phosphorylation (Haghighi et al., 2006). Interestingly, when the wild-type phospholamban got removed by mating the transgenic mice with the *Pln* knockout mice, hearts are hypercontractile and show a progression to cardiac hypertrophy (Chu et al., 1998; Haghighi et al., 2012). More importantly, mutant phospholamban did not colocalize with SERCA2a and is localized instead at the plasma membrane and alters function of the Na/K ATPase (NKA). Taken together, the p.R14del mutation has a complex effect on SR calcium homeostasis, impacting both PLN inhibition of SERCA and PKA-mediated phosphorylation of PLN via the beta-adrenergic pathway. In addition, a link between *PLN* mutations, disturbed calcium handling, and the intercalated discs has been postulated. The *PLN*-p.R14del mutation results in an accumulation of diastolic calcium, which, in turn, would be able directly or via an activation of Ca^2+^ sensitive proteins, such as calmodulin-dependent kinase II (CaMKII) and calcineurin (CnA), to affect the maladaptive remodeling of the macromolecular protein complex that forms the intercalated disc (van Opbergen et al., 2017). *PLN*-p.R14del may be a good example for a phenotype of arrhythmogenic dilated cardiomyopathy covering both entities of DCM and ACM.

### Animal Models for ACM Genes Encoding Proteins of the Nuclear Envelope

Proteins of the nuclear envelope, which surround the nucleus and are continuous with the sarcoplasmic reticulum in the cell, are usually highly conserved and ubiquitously expressed. They have many distinct functions and are involved in the pathology of the heart and other organs. They contribute to the assembly of orderly structures and stability and strength of cells and participate in the regulation of DNA replication and transcription through chromatin structures (Stroud, 2018). Human mutations in *TMEM43*, *LMNA*, and *LEMD2* have been associated with often highly penetrant, aggressive disease with respect to ventricular arrhythmia and sudden cardiac death. In addition, the human phenotype mainly affects the left or both ventricles, whereas a predominant right ventricular involvement appears unusual.

#### Transmembrane Protein-43

The transmembrane protein-43 (TMEM43) localizes in the inner nuclear membrane, where it interacts with lamin and other proteins of the linker of nucleoskeleton and cytoskeleton (LINC) complex (Bengtsson and Otto, 2008). The best characterized human *TMEM43* missense mutation causing autosomal-dominant inherited ACM (#MIM 604400) is p.S358L. *TMEM43*-p.S358L generates a severe sex-influenced lethal ACM with left ventricular dilation, fibro-fatty replacement of cardiomyocytes, heart failure, and early death, especially in male patients (Merner et al., 2008; Hodgkinson et al., 2013; Milting et al., 2015). Currently, the exact molecular mechanism for earlier presentation and decreased life expectancy in males is unknown because *TMEM43* is not localized on a gonosome. Information about the role of TMEM43 in health and disease *in vivo* have been assessed just very recently in different mouse models. The first two mouse models have globally knocked out murine *Tmem43* as well as introduced the corresponding human mutation p.S358L via CRISPR/Cas9 knock-in into *Tmem43* (Stroud et al., 2018). Surprisingly, both mouse models did not show a cardiac phenotype at baseline and after pressure overload. In contrast, a very similar *Tmem43* knock-in mouse model carrying the p.S358L mutation, generated by traditional homologous recombination-mediated genomic targeting, shows features of cardiomyopathy with fibro-fatty infiltration but preserved cardiac function. The authors show activation of NFκB/TGFβ signaling, which may cause the fibrotic changes (Zheng et al., 2019). More importantly, cardiac-specific overexpression of human *TMEM43*-p.S358L in a transgenic model recapitulates the severe human phenotype. Transgenic hearts show severe biventricular cardiac dysfunction, fibro-fatty replacement of the myocardium, and decreased survival. Conduction defects precede the structural abnormalities. TMEM43-p.S358L showed partial delocalization to the cytoplasm and reduced interaction with emerin and β-actin, suggesting that a reduced interaction between the nucleus and the cytoskeleton contributes to cardiomyocyte death under biomechanical stress. Inhibition of the GSK3β pathway improved cardiac function (Padron-Barthe et al., 2019). However, a lot of questions still remain open, for example, with regards to the sex-specific effect and the mechanism of arrhythmia preceding the structural changes.

#### Lamin A/C

*LMNA* encodes nuclear proteins lamin A and C, which are widely expressed IF proteins within the inner nuclear membrane and a relatively common cause of familial DCM (Stroud, 2018). Cardiac manifestations are also part of isolated cardiomyopathy or associated with other syndromic features, ranging from skeletal myopathies, lipodystrophy, and neuropathy to premature aging. Patients with *LMNA* mutations affecting the heart present with conduction disease and ventricular arrhythmia, often preceding left and sometimes biventricular dysfunction. However, screening of typical ACM patients identified *LMNA* mutation carriers with severe right ventricular involvement, sudden cardiac death, and conduction abnormalities, suggesting a genetic overlap of DCM and ACM (Quarta et al., 2012; Valtuille et al., 2013). Mouse models carrying several missense mutations have been generated (Chen et al., 2019); however, a particular mutation with an overlapping phenotype to ACM has not been reported. More generally, homozygous *Lmna* knockout mice showed severely retarded postnatal growth with the appearance of muscular dystrophy and DCM and died by about 8 weeks of age mimicking a phenotype of Emery–Dreifuss muscular dystrophy, DCM, and progeria (Sullivan et al., 1999; Nikolova et al., 2004). However, human DCM is an autosomal-dominant disease and, therefore, heterozygous mice for *Lmna+/-* have been further evaluated and which demonstrate atrioventricular (AV) conduction defects, atrial and ventricular arrhythmia, and at an advanced age also impaired cardiac contractility, which mimics haploinsufficiency of human disease (Wolf et al., 2008). Knockdown of lamin during zebrafish development shows also a cardiomyopathic phenotype with significant bradycardia (Vogel et al., 2009), whereas another zebrafish model in which cryptic splicing of *lmna* generated a deletion of eight amino acids revealed embryonic senescence and S-phase accumulation/arrest as well as abnormal muscle and lipodystrophic phenotypes (Koshimizu et al., 2011). Finally, the underlying pathogenesis for *LMNA* mutations causing cardiac features is complex and part of a multisystem disease pathology. The type of *LMNA* mutation may modify the risk of disease progression and arrhythmia; however, many human missense mutations appear to be extensively heterogeneous, but in particular, truncating mutations seem to have a higher risk for arrhythmia and sudden cardiac death (van Rijsingen et al., 2012).

#### LEM-Domain Containing Protein-2

More recently a human homozygous missense mutation (p.L13R) in another inner nuclear membrane protein, encoding the LEM-domain containing protein-2 (LEMD2), has been found to cause an ACM with juvenile cataract in the Hutterite population. The cardiac phenotype is characterized by ventricular arrhythmia and sudden cardiac death, which often precedes dilation and left-ventricular dysfunction (Abdelfatah et al., 2019). Interestingly, mutations in the same gene but a different domain cause a progeria-like phenotype (Marbach et al., 2019). So far, there is no mouse model simulating this specific mutation, but the global knockout of *Lemd2* in mice is embryonically lethal at E11.5 and shows reduced size of most tissues with thin myocardium and underdeveloped trabeculae, suggesting an importance of *Lemd2* during mouse development (Tapia et al., 2015). More specific data about the role of LEMD2 in the development of cardiomyopathy are required to explain the clinical phenotype and its pathology, which appears not to be neither classical for DCM nor ACM and may present an overlap of both forms.

### Animal Models for ACM Genes Encoding Z-Band Proteins

Z-bands are multiple-protein structures responsible for anchoring thin sarcomeric filaments as well as the giant sarcomere protein titin (Luther, 2009) and impact the structural integrity of the sarcomeres. Mutations in several genes involved in Z-band formation are involved in genetic cardiomyopathies, including ACM (Gerull et al., 2019).

#### Desmin

Although desmin is not directly a Z-band protein, it forms cytoplasmic IF and is involved in connection of the Z-bands (Hijikata et al., 1999), desmosomes (Choi et al., 2002), mitochondria (Capetanaki, 2002), and also the nuclei (Heffler et al., 2020) in cardiomyocytes. Mutations in *DES* cause different skeletal and cardiac myopathies, including DCM (Li et al., 1999; Brodehl et al., 2016a), HCM (Harada et al., 2018), RCM (Brodehl et al., 2019c), LVNC (Marakhonov et al., 2019; Kubanek et al., 2020), and also ACM (Klauke et al., 2010; Bermudez-Jimenez et al., 2018). Desmin consists of a central α-helical rod domain flanked by nonhelical head and tail domains. The majority of pathogenic *DES* mutations are heterozygous missense or small in-frame deletion mutations spread over the whole rod domain (Brodehl et al., 2018). Many *DES* mutations disturb dominantly the filament assembly and cause an abnormal cytoplasmic desmin aggregation (Brodehl et al., 2012). In the 1990s, two groups independently generated *Des*-deficient mice models (Li et al., 1996; Capetanaki et al., 1997). *Des*-deficient mice develop severe fibrosis, cardiac calcification, and biventricular cardiomyopathy (Psarras et al., 2012). Correspondingly, knockdown of *desma* and *desmb* causes cardiac edema and structural disorganized muscles in zebrafish (Li et al., 2013). However, the major pathomechanism consisting of abnormal desmin aggregation is not modeled by this LOF animal model. In contrast, the Des*-*p.R349P knock-in mice developed toxic desmin aggregates and presented mitochondrial defects (Clemen et al., 2015; Winter et al., 2016; Stockigt et al., 2020). These mice developed skeletal myopathy in combination with DCM, including cardiac arrhythmia and conduction defects. In 2015, Ramspacher et al. inserted fluorescence proteins at position 460 in *desma* of the zebrafish (Ramspacher et al., 2015). These zebrafish developed cardiac arrhythmia and cardiac dysfunction, leading to decreased viability. In conclusion, desmin animal models suited for modeling of LOF and toxic dominant desmin accumulation are available and partially mimic human ACM.

#### LIM Domain Binding Protein-3

Mutations in *LDB3*, encoding LIM domain binding protein-3, which is also known as cipher or ZASP, cause myofibrillar myopathy and different cardiomyopathies in humans (Vatta et al., 2003; Selcen and Engel, 2005). ZASP binds to α-actinin-2 and is important for the structural integrity of the Z-bands (Lin et al., 2014). In 2015, Lopez-Ayala and coworkers identified the novel heterozygous missense mutation *LDB3-*c.1051A > G in the genome of an ACM index patient using a next-generation sequencing (NGS) gene panel. Cascade genetic screening revealed cosegregation of this mutation within the family supporting its pathogenicity (Lopez-Ayala et al., 2015). In 2009, Zheng et al. developed conditional cardiac-specific and inducible cardiac-specific *Ldb3* deficient mouse models (Zheng et al., 2009). Both mouse models presented severe biventricular dilation and cardiac dysfunction, leading to increased mortality. Of note, the Z-bands were highly disorganized in cardiomyocytes from *Ldb3-*deficient mice, and the ERK/Stat3 signaling was altered (Zheng et al., 2009). In addition, a transgenic mouse model with cardiac-specific overexpression of mutant LDB3-p.S196L was generated (Li et al., 2010). These mice developed DCM in combination with cardiac conduction defects and ventricular arrhythmia associated with ultrastructural defects of the sarcomeres.

#### α-Actinin-2

Recently, a novel missense mutation p.Y473C was identified in *ACTN2*, encoding α-actinin-2, in a family with left-dominant ACM (Good et al., 2020). Pathogenic mutations in *ACTN2* have been also identified in patients with HCM, DCM, and LVNC, indicating a genetic overlap of associated phenotypes (Mohapatra et al., 2003; Bagnall et al., 2014; Girolami et al., 2014). α-Actinin-2 anchors actin filaments and titin to the Z-bands. Gupta et al. knocked down *actn2* in zebrafish using morpholino oligonucleotide injections, revealing skeletal and cardiac muscle defects (Gupta et al., 2012). However, there is currently no specific mouse or zebrafish model available for ACM-associated human *ACTN2* mutations.

### Animal Models for ACM Genes Encoding Proteins Involved in Cell–Cell and Cell-ECM Adhesion

Mutations in five genes (*CTNNA3*, *CDH2*, *TJP1*, *ILK*, *FLNC*) encoding proteins involved in cell–cell or ECM interaction are associated with human ACM (Figure 1). However, because these mutations have been discovered recently, the amount and impact for ACM is currently inexplicit. Nevertheless, for some of them, specific animal models provide additional evidence for their pathogenicity (Figure 4).

#### αT-Catenin

Rampazzo’s group identified a *CTNNA3* missense and a small in-frame 3-bp deletion in two ACM families (van Hengel et al., 2013). However, it seems that *CTNNA3* mutations are rare (Christensen et al., 2011; Gandjbakhch et al., 2013). *CTNNA3* encodes αT-Catenin, which is a structural protein involved in the area composita. Of note, conditional *Cttna3* knockout mice were viable and fertile but developed progressive cardiomyopathy, including left-ventricular dilation and decreased ejection fraction (Li et al., 2012). Despite that, the relevance of *CTNNA3* for ACM or other cardiomyopathies has to be investigated in more detail.

#### N-Cadherin

Recently, two independent groups identified *CDH2* missense variants (p.D407N and p.Q229P) in the genome of ACM patients (Mayosi et al., 2017; Turkowski et al., 2017; Figure 1). Cosegregation analyses within the families and absence in population databases might indicate pathogenicity of these *CDH2* mutations. Similar to the desmosomal cadherins, N-cadherin is localized in multiple-protein complexes in the intercalated disc, which are called adherens junctions or area composita (Franke et al., 2006; Zhao et al., 2019). The relevance of N-cadherin for ACM is additionally supported by two different mouse models (Figure 4). Cardiac-specific overexpression of chicken *CDH2* caused biventricular cardiomyopathy in combination with increased expression of stress markers and a decreased connexin-43 expression (Ferreira-Cornwell et al., 2002). Similarly, cardiac-specific inducible *Cdh2* knockout mice developed DCM in combination with ventricular arrhythmia and conduction abnormalities, leading to premature death after 2 months (Kostetskii et al., 2005; Li et al., 2005). Although no functional data about human *CDH2* mutations were currently available, both mouse models underline the impact of N-cadherin for normal heart function *in vivo*. However, future studies are needed to determine the pathomechanism of *CDH2* mutations at the molecular level.

#### Tight Junction Protein-1

Currently, there is one single report describing four different *TJP1* missense mutations in ACM patients (De Bortoli et al., 2018). *TJP1* encodes tight junction protein-1 localized at the intercalated disc, which is also known as zonula occludens-1 (ZO1). Global knockout of *TJP1* caused embryonic lethality in mice, limiting functional analyses (Katsuno et al., 2008). Because of the limited knowledge about the relevance of tight junction protein-1 in ACM, cardiac-specific conditional knockout or knock-in animal models might contribute to clinical interpretation of *TJP1* mutations in future.

#### Integrin-Linked Kinase

Recently, we identified two different missense mutations in *ILK* in the genome of ACM patients by NGS. One of these mutations occurred *de novo* (Brodehl et al., 2019d). Before, *ILK* mutations have been described for DCM patients (Knoll et al., 2007). *ILK* encodes integrin-linked kinase. There is still an ongoing debate about whether ILK is a real or pseudo kinase involved in linkage to the integrin system (Hannigan et al., 2011; Ghatak et al., 2013; Vaynberg et al., 2018). Transgenic zebrafish with a cardiac-specific overexpression of mutant ILK presented a decreased fractional shorting and showed premature death (Brodehl et al., 2019d). Correspondingly, a spontaneous homozygous *ilk* mutation (p.L308P) caused cardiac edema, decreased cardiac function, and premature death in the zebrafish (Bendig et al., 2006; Pott et al., 2018). Muscle-specific *Ilk* knockout mice showed an increased mortality, ventricular dilation, arrhythmia, and cardiac fibrosis (White et al., 2006; Quang et al., 2015). In addition, Akt phosphorylation was reduced, and connexin-43 was downregulated in these mice.

#### Filamin-C

Mutations in *FLNC* were initially identified in patients with myofibrillar myopathy (MFM) (Vorgerd et al., 2005; Shatunov et al., 2009). *FLNC* encodes filamin-c, which is a huge cytolinker protein involved in linkage of the area composita, costameres, and actin filaments. The relevance of *FLNC* mutations for development of different cardiomyopathies, such as HCM (Valdes-Mas et al., 2014), RCM (Brodehl et al., 2016b), and DCM (Begay et al., 2016; Nozari et al., 2018) has been more recently recognized. In addition, mutations in *FLNC* cause ACM (Begay et al., 2018; Hall et al., 2019, 2020; Augusto et al., 2020; Brun et al., 2020). Global or cardiac-specific *Flnc* deficiency was embryonically lethal in mice, limiting the functional analyses (Zhou et al., 2020). Mice expressing a mutant C-terminal truncated form of filamin-C died perinatal by respiratory failure, but no detailed cardiac analyses have been performed (Dalkilic et al., 2006). Therefore, Chen’s group developed inducible cardiac-specific *Flnc-*deficient mice. These mice showed premature death, increased cardiac stress marker expression, cardiac fibrosis, and cardiac dysfunction. Interestingly, several proteins of the costameres, the intercalated discs, or the IFs were highly increased (Zhou et al., 2020). In summary, there is evidence from human and animal data that demonstrate the impact of filamin-C for heart function. However, the specific impact of *FLNC* mutations for ACM needs further investigation in the future.

### Animal Models for ACM Genes Involved RNA Binding and Processing

Mutations in *RBM20*, encoding the RNA-binding motif protein 20 (RBM20), have been recently identified in patients with ACM (van den Hoogenhof et al., 2018; Parikh et al., 2019). Initially, pathogenic missense mutations in *RBM20* were described in DCM (Brauch et al., 2009) and LVNC patients (Sedaghat-Hamedani et al., 2017). RBM20 is a splicing factor and consists of one N-terminal leucine-rich domain, two zinc finger domains, a RNA recognition motif, an arginine- and serine-rich (RS) domain and a glutamate-rich region (Watanabe et al., 2018). The majority of pathogenic *RBM20* mutations is localized in the RS domain although also mutations in the glutamate-rich region have been described (Beqqali et al., 2016). Using a spontaneous rat model carrying a homozygous large deletion affecting *rbm20*, Guo *et al. re*vealed that Rbm20 is involved in cardiac splicing of several genes, including *ttn* (titin) and *ldb3*. Of note, several of the known targets of Rbm20, such as *RYR2*, are ACM-associated genes. Maatz et al. determined further RBM20 target genes, such as *Myh7*, *Nexn*, *Tnnt2*, and *Ryr2* (Maatz et al., 2014). The *Rbm20*-deficient mice presented cardiomyopathy in combination with arrhythmia, leading to increased mortality. This phenotype in rats is in good agreement with the phenotype of cardiac-specific *Rbm20*-deficient mice, which developed arrhythmogenic DCM (Khan et al., 2016; van den Hoogenhof et al., 2018). Interestingly, adenovirus-mediated Rbm20 overexpression rescued the *ttn* splicing defect in *rbm20*-deficient cardiomyocytes, underlining a putative therapeutic strategy (Guo et al., 2012). Murayama and colleagues generated a knock-in mouse model carrying Rbm20-p.S647A in the RS domain. Although the detailed phenotype of these mice has yet not been described in detail, the authors demonstrated defects in splicing of titin in the murine heart (Murayama et al., 2018). Interestingly, mutations in the RS domain prevent nuclear transport of RBM20 (Murayama et al., 2018; Brodehl et al., 2019b). Another ACM-associated gene encoding a putative RNA-binding protein (Han et al., 2019) might be *SORBS2*, encoding sorbin and SH3 domain-containing protein-2 (Ding et al., 2019). The exact molecular function of *SORBS2* is currently unknown. However, in a preprinted manuscript, Ding et al. described several novel human *SORBS2* variants in ACM patients and generated cardiac-specific *Sorbs2*-deficient mice. These mice develop biventricular cardiomyopathy, leading to an increased mortality (Ding et al., 2019). However, it is currently unclear if SORBS2 is a RNA-binding protein or a desmosomal protein. Currently, it is difficult to predict the relevance of *SORBS2* in ACM.

### Further Animal Models for Human ACM-Associated Genes

Few additional genes have been reported in association with ACM either as part of an overlap phenotype with other cardiomyopathies (*TTN*) or where the genes have been suggested as a candidate gene for ACM (*TGF*β*3*) or as part of a complex syndrome (*TP63*). Their overall importance for the disease remains elusive.

#### Titin

Truncating mutations (TTNtv) in the sarcomeric gene *TTN* have been reported with a frequency of approximately 25% as the most common cause of DCM (Herman et al., 2012; Roberts et al., 2015). However, their role for other cardiomyopathies remains elusive (Gerull, 2015). Moreover, missense variants, which are predicted to be deleterious, are found in about 7% of DCM patients, which was comparable with the frequency in the reference population, suggesting that their causative relevance for DCM is not established (Akinrinade et al., 2019). For ACM, two studies have been published in which exclusively *TTN* missense variants have been suggested as a cause of ACM (Taylor et al., 2011; Brun et al., 2014), which might be, under current view, questionable although a modifying effect cannot be excluded.

Interestingly, in particular, the zebrafish has been used to mimic different TTNtv, but also a mouse model has been developed, which corresponds to a human A-band truncation mutation (Gerull et al., 2002). Mice homozygous for the truncation are embryonically lethal at E9.5 due to defects in the sarcomere formation, whereas heterozygous animals have no cardiac abnormalities at baseline but develop fibrotic remodeling under hemodynamic stress (Gramlich et al., 2009). Using different genome-editing approaches, truncation mutations have been introduced and analyzed at different locations of the zebrafish *ttn* gene (Zou et al., 2015; Shih et al., 2016; Huttner et al., 2018). Homozygous truncations at the N-terminal and C-terminal molecule caused severe defects in cardiac contractility, and the zebrafish died within 2 weeks. Interestingly, C-terminal truncations led to severe skeletal muscle myopathies but not N-terminal. A newly discovered internal promotor produces the so-called C-terminal titin isoform “Cronos,” which is translated into a protein and explains the differences between the phenotypes, depending on the location of the truncation with or without a subsequent disruption of the “Cronos” isoform (Zou et al., 2015). Others also modeled truncation mutations in zebrafish and supported the exon usage hypothesis (Shih et al., 2016), which was previously suggested based on human studies (Roberts et al., 2015). The impact of heterozygous *ttn* truncations at an adult age was further investigated. Like in humans, heterozygous truncations led to spontaneous DCM with reduced baseline ventricular systolic function and failed to mount a hypercontractile response when challenged by hemodynamic stress (Huttner et al., 2018).

#### Transforming Growth Factor-ß3

The first genomic locus for ACM at 14q23-q24 (ARVD1; #MIM 107970) was discovered by linkage analysis in Italian families, but a pathogenic mutation in exonic sequences of several candidate genes was not detected (Rampazzo et al., 2003). Later on, two genetic variants in the untranslated regions of the transforming growth factor-β3 (TGFβ3) were identified; however, the role of *TGF*β*3* as a causative gene for ACM (Beffagna et al., 2005) remains still open. Moreover, *Tgf*β*3* knockout mice showed no specific cardiac phenotype but suffer from abnormal lung development and cleft palate at birth (Kaartinen et al., 1995; Proetzel et al., 1995). On the other hand, TGFβ signaling has been suggested to play a role in the disease process of ACM and has been shown to be activated in the pathogenesis of *TMEM43*-related disease as well as for mutant desmosomal proteins, such as plakoglobin and desmoplakin (Li et al., 2011; Giuliodori et al., 2018; Zheng et al., 2019).

#### Tumor Protein-63

Another interesting protein is the tumor protein p63 (*TP63*). Mutations in *TP63* can cause a range of overlapping syndromic ectodermal dysplasias (EDs). In some cases of EDs, a congenital heart defect was found; however, a case report described a missense mutation in *TP63* in a patient with ED and typical cardiac findings of ACM (Valenzise et al., 2008). More recently, a novel heterozygous nonsense variant was reported in a typical nonsyndromic ACM case, suggesting that haploinsufficiency may play a role in the disease process (Poloni et al., 2019). Knockout of *Tp63* in mice and zebrafish are viable and indicate its role in development and ectodermal differentiation, but no obvious cardiac abnormalities have been detected in either homozygous or heterozygous animals (Mills et al., 1999; Santos-Pereira et al., 2019). A potential role for *TP63* as an ACM associated gene needs to be further defined.

## ACM Animal Models Without Human Genetic Correlation

Several animal models have been described for which the responsible gene is still unknown or no human genetic mutations have been associated with ACM. Asano et al. generated a spontaneous mouse model with massive right-ventricular fibrosis, calcification, and prolonged QRS duration with recessive inheritance. Linkage analyses revealed a 1031-bp retroposon in the *Rpsa* gene. *Rpsa* or *Lamr1* encodes the laminin receptor-1, which is also called ribosomal protein SA. Transgenic mice with cardiac-specific or systemic overexpression of this mutant form of the laminin receptor 1 caused ACM, supporting the pathogenicity of this mutant gene (Asano et al., 2004). In humans, heterozygous mutations in *RPSA* cause isolated congenital asplenia by haploinsufficiency (MIM, #271400) (Bolze et al., 2013).

Since the 1980s, it has been recognized that ACM occurred in Boxer dogs with similar clinical signs to humans, including ventricular arrhythmia, ventricular dilation, and fibro-fatty replacement of the myocardium (Miller et al., 1985; Harpster, 1991; Basso et al., 2004). Nearly all mouse and zebrafish models are unable to display fibro-fatty replacement of myocardial tissue, which is a typical hallmark of human ACM. In contrast, the Boxer ACM model is also able to mimic those pathological findings. In addition, inflammatory cell infiltration and apoptosis were present in this large-animal model (Basso et al., 2004). Meurs et al. analyzed pedigrees of Boxer dogs and suggested an autosomal dominant inheritance (Meurs et al., 1999). Based on a genome-wide association study, a deletion in the untranslated region of *STRN*, encoding striatin, was suggested as the pathogenic mutation causing Boxer ACM (Meurs et al., 2010). However, Cattanach et al. demonstrated that this *STRN* mutation is not causative for Boxer ACM, but might be genetically linked to the responsible gene on chromosome 17 (Cattanach et al., 2015). Currently, no *STRN* mutation for human ACM patients is listed in the HGMD or the ARVD/C Genetic Database^4^. In addition, ACM was described for English Bulldogs and Weimaraners (Eason et al., 2015; Cunningham et al., 2018). Corresponding to Boxer ACM, it was shown that ACM presented with right-ventricular dilation, thin right-ventricular wall diameter, ventricular tachycardia, and severe fibro-fatty replacement of the myocardium, which occurred spontaneously in domestic cats (Fox et al., 2000; Harvey et al., 2005; Ciaramella et al., 2009). However, the genetic mutations responsible for ACM in cats is currently unknown.

## Summary and Outlook

Because the first disease genes of ACM were discovered more than 20 years ago, a substantial number of *in vivo* models have been generated and characterized to fill the important gap between the underlying genetic defect and the observed clinical phenotype. Those models often recapitulate at least partially pathological features of ACM and gain mechanistic insights into the disease pathogenesis with the opportunity to develop targeted therapies in the future. However, a past *in vivo* model strategy mimics LOF much better due to targeted knockouts of affected proteins, but it is limited in modeling missense mutations, often leading to a gain-of-function or toxic effect. Transgenic models used for missense mutations still mainly overexpress the human mutant proteins, which often have different effects in mice compared to the human phenotype. Knock-in animal models represent a much better system for studying human missense mutations as the mutation is inserted into the endogenous gene and works under the control of its own promotor. Nevertheless, even if the human disease mutation is conserved, it is still kept in mind that substantial differences exist between the human heart and mice or fish hearts. In particular in ACM, the hallmark of fatty tissue in the human right ventricle is almost never seen in the corresponding model system. From recent advances in genetic technology emerged the CRISPR/Cas9 genome-editing approach, which has simplified the generation of knockout and knock-in models and will become the technology of choice for studying human gene mutations in the future.

## Author Contributions

BG and AB contributed to the writing and visualization. Both authors agreed to be accountable for the content of this work.

## Conflict of Interest

The authors declare that the research was conducted in the absence of any commercial or financial relationships that could be construed as a potential conflict of interest.

## Footnotes

1 https://www.proteinatlas.org/

2 https://portal.biobase-international.com/hgmd/

3 https://www.proteinatlas.org/ENSG00000104881-PPP1R13L/tissue

4 https://molgenis136.gcc.rug.nl
