# Supplementary material for: Genetic Animal Models for Arrhythmogenic Cardiomyopathy
Source: Front Physiol. 2020 Jun 24;11:624. doi: 10.3389/fphys.2020.00624 (PMC7327121; doi:10.3389/fphys.2020.00624)
Supplement: Supplementary file 1 [file Data_Sheet_1.docx]

**Supplementary Tables**

**Table 1.** Overview about rodent models for ACM.

| Protein (Gene) | Rodent Model | Major Finding | Reference |
| --- | --- | --- | --- |
| Plakoglobin (*JUP*) | Global *Jup* knock-out (homozygous) | - Embryonic lethal - Severe heart defects - Skin blistering - Reduced number of desmosomes | (Bierkamp et al., 1996) |
|  | Global *Jup* knock-out (homozygous) | - Embryonic lethal - Severe heart defects - Reduced number of desmosomes | (Ruiz et al., 1996) |
|  | Global *Jup* knock-out (heterozygous) | - Right ventricular dilation - Reduced right ventricular function - Arrhythmia - No cardiac structural abnormalities - Endurance training increased the cardiac phenotype | (Kirchhof et al., 2006;Fabritz et al., 2011) |
|  | Conditional cardiac specific *Jup* knock-out (*Myh6*:*Cre*) | - Ventricular dilation - Ventricular arrhythmia - Severe cardiac fibrosis - Severe fibrotic scars / aneurysm - Absence of cardiac desmosomes - Increased TGFβ signalling | (Li et al., 2011a) |
|  | Homozygous 2-bp deletion knock-in mouse (deletion in exon 11 leading to premature termination codon) | - Perinatal lethality - Decreased expression of mutant plakoglobin - No ventricular fibrosis | (Zhang et al., 2015) |
|  | Homozygous 2 bp deletion knock-in mouse (deletion in exon 11 leading to premature termination codon) and removal of the introns between exon10-14 | - Removal of the introns blocks nonsense mediated RNA-decay leading to an expression of truncated plakoglobin comparable to wild-type plakoglobin - No phenotype |  |
|  | *Myh6:3xFLAG-Jup-*WT, transgenic mouse | - Increased mortality - Fibrosis | (Lombardi et al., 2011) |
|  | *Myh6:3xFLAG-Jup-*c.2037-2038delTG, reported as *Jup*^23654del2^, transgenic mouse | - Increased mortality - Fibrosis - Reduced membrane localization of mutant plakoglobin |  |
|  | Conditional, inducible, cardiac specific *Jup* knock-out *(Myh6:MerCreMer)* | - Cardiac dysfunction - Inflammation - Replacement fibrosis - Decreased expression of desmosomal proteins - Activation of β-Catenin signaling | (Li et al., 2011b) |
| Ryanodine Receptor-2 (*RYR2*) | *Ryr2* (intron after exon1), global knock-out, homozygous | - Embryonically lethal E10 - Morphological abnormalities in the heart tube - Large vacuolate sarcoplasmic reticulum (SR) - Structurally abnormal mitochondria | (Takeshima et al., 1998) |
|  | *Ryr2* flox/flox :(Myh6:MerCreMer) mice, conditional c*Ryr2* KO, induced at 8-20 weeks of age | - Acute approximately 50% loss of RYR2 protein in the heart - Functional and structural hallmarks of heart failure, including sudden cardiac death - Fatal arrhythmia | (Bround et al., 2012) |
|  | *Ryr2-p.*R176Q^+/-^ targeted knock-in, heterozygous | - Reduced right ventricular end-diastolic volumes, but normal RV structure - Ventricular tachycardia after injection of caffeine and epinephrine - Increased incidence of spontaneous Ca^2+^ oscillations in single cardiomyocytes in the absence and presence of isoproterenol | (Kannankeril et al., 2006) |
|  | *Ryr2*-p.L433P^+/-^  Knock-in, heterozygous | - Atrial fibrillation, induced by atrial burst pacing in 5 of 14 mice - Leaky calcium channels in the sarcoplasmic reticulum of atrial myocytes | (Shan et al., 2012) |
|  | *Ryr2*-p.N2386I^+/-^  Knock-in, heterozygous | - Atrial fibrillation, induced by atrial burst pacing in 9 of 15 mice - 1,4-benzothiazepine (Rycal S107) prevents atrial burst pacing-induced atrial fibrillation - Leaky calcium channels in the sarcoplasmic reticulum of atrial myocytes |  |
| Desmoplakin (*DSP*) | Global *Dsp* knock-out (homozygous) | - Embryonic lethal | (Gallicano et al., 1998) |
|  | *Myh6*:*DSP*-p.R2834H-FLAG, transgenic mouse | - Cardiac fibrosis - Cardiomyocyte apoptosis - Ventricular dilation - Cardiac dysfunction - Ultrastructural changes of the intercalated disc | (Yang et al., 2006) |
|  | *Myh6*:*DSP*-WT-FLAG, transgenic mouse, control | - No structural, morphological or functional alterations |  |
|  | Conditional cardiac specific *Dsp* knock-out (*Myh6:Cre; ∆exon2*) | - High lethality of homozygous embryos (homozygous) - Decrease postnatal survival rate after birth (homozygous) - Severe cardiac fibrosis (heterozygous and homozygous) - Decreased fractional shortening (heterozygous) - Ventricular tachycardia (heterozygous) - Exercise might has beneficial impact (heterozygous) - Increased expression of inhibitors of the *Wnt*-pathway (heterozygous) - Activation of epithelial-mesenchymal transition (heterozygous) - Inflammation (heterozygous) | (Garcia-Gras et al., 2006;Cheedipudi et al., 2019) |
|  | Conditional cardiac specific *Dsp* knock-out (*Myh6:Cre*), heterozygous | - Normal echocardiogram - Delayed conduction - Inducible ventricular tachycardia - Reduced connexin-43 expression | (Gomes et al., 2012) |
|  | Conditional cardiac specific *Dsp* knock-out (*Mlc2v:Cre*), homozygous | - Biventricular cardiomyopathy - Ultrastructural defects - Fibro-fatty replacement of the myocardium - Conductions defects - Decreased connexin-40 and -43 expression - Increased mortality after birth | (Lyon et al., 2014) |
|  | Homozygous, spontaneous 10 bp insertion mutation 🡪 frameshift leading to premature termination codon | - Abnormal coat - Epidermal blistering - Ventricular fibrosis - Electrophysiological abnormalities | (Herbert Pratt et al., 2015) |
|  | Conditional cardiac conduction specific *Dsp* knock-out (*Hcn4*:Cre) | - Increased sinus pauses | (Mezzano et al., 2016) |
| Plakophilin-2 (*PKP2*) | Global *Pkp2* knock-out (homozygous) | - Embryonic lethality - Reduced trabeculation - Abnormal cytoplasmic desmoplakin aggregation - Ruptures of the cardiac walls - Blood leakage | (Grossmann et al., 2004) |
|  | Global *Pkp2* knock-out (heterozygous) | - Decreased plakophilin-2 expression (haploinsufficiency) - Altered electrophysiology - No histological differences - Ultrastructural defects | (Cerrone et al., 2012;Leo-Macias et al., 2015) |
|  | *Myh6*:*Pkp2*-p.S329X-T2A-Ruby, transgenic mouse | - No fibro-fatty replacement - Ultrastructural defects, intercalated disc ruptures - Structural and electrophysiological abnormalities - Decreased expression of other desmosomal proteins - Remodelling of connexin-43 and sodium channels - Electrophysiological anomalies | (Moncayo-Arlandi et al., 2016) |
|  | Conditional, inducible, cardiac specific *Pkp2* knock-out (*Myh6*:*Cre*-ER(T2); ∆exons 2-3) | - Decreased left ventricular ejection fraction - Decrease survival - Severe cardiac fibrosis - Arrhythmia - Remodeling of proteins involved in Ca^2+^ signaling | (Cerrone et al., 2017) |
|  | Adeno-associated virus (AAV) mediated expression of *PKP2*-p.R735X | - Exercise in combination with expression of PKP2-p.R735X leads to right ventricular dysfunction | (Cruz et al., 2015) |
| Transforming Growth Factor β3 (*TGFβ3*) | Global *Tgfb3* knock-out, homozygous, heterozygous no phenotype | - Abnormal lung development and cleft palate in newborn mice - Defects of epithelial-mesenchymal interaction - No specific cardiac phenotype | (Kaartinen et al., 1995;Proetzel et al., 1995) |
| Desmocollin-2 (*DSC2*) | Conditional *Dsc2* knock-out *(Cre-*deleter*,* ∆exon 2) | - Viable and no obvious phenotype - Normal desmosomes - Decreased cardiac stress resistance |  |
|  | *Myh6*:*DSC2*-HA, transgenic mouse | - Severe fibrotic scars - Severe biventricular dysfunction - Fibrosis, necrosis and calcification - Cardiac aseptic inflammation | (Brodehl et al., 2017) |
|  | *Dsc2*-p.G790del (knock-in) | - No right ventricular structural/mechanical defects - No arrhythmia - Slight contractile dysfunction and Ca^2+^ dysfunction | (Hamada et al., 2020) |
| Desmoglein-2 (*DSG2*) | Global *Dsg2* knock-out (loss of exon 7‑8) | - Embryonic lethal | (Eshkind et al., 2002) |
|  | *Myh6*:*Dsg2*-p.N271S, transgenic mouse | - Widening of the intercalated disc - Arrhythmia - *In vivo* interaction between Dsg2 and Na_v_1.5 | (Pilichou et al., 2009;Rizzo et al., 2012) |
|  | *Myh6:Dsg2*-WT transgenic mouse, control | - No abnormalities |  |
|  | In-frame deletion of *Dsg2* Exon 4-6 (parts of extracellular domains 1 and 2 are deleted) | - Cardiac fibrosis, necrosis, calcification - Increased expression of cardiac stress markers - Cardiac insufficiency - Fibrotic scars - Ventricular arrhythmia - Cardiac aseptic inflammation - Reduced number of desmosomes - Widening of the intercalated disc | (Krusche et al., 2011;Kant et al., 2012;Buck et al., 2018) |
|  | Conditional cardiac specific *Dsg2* knock-out (*Myh6*:Cre) | - Ventricular dilation - Calcification - Cardiomyocytes necrosis - Aseptic inflammation - Replacement fibrosis - Absence of cardiac desmosomes | (Kant et al., 2015) |
|  | *Myh6:DSG2-*p.Q558X-FLAG, transgenic mouse | - Fibrosis - Reduced number of desmosomes - Decreased Wnt/β-catenin signaling - Increased miRNA expression signatures | (Calore et al., 2019) |
|  | *Myh6:DSG2-*WT-FLAG*,* transgenic mouse, control | - No obvious phenotype ∆ |  |
|  | Conditional *Dsg2* knock-out (*CMV:Cre;* ∆exon 4-5) | - Decreased fractional shortening and ejection function - Severe cardiac fibrosis - Loss of plakoglobin and connexion-43 localization at the intercalated disc - Inflammation - Arrhythmia - Improvement of left ventricular function by GSK3β inhibition | (Chelko et al., 2016) |
| Transmembrane Protein-43 (*TMEM43*) | *Tmem43* knock out  Sox2-Cre deleter mice | - Normal cardiac function - Normal response to transverse aortic constriction - Normal localization and expression of LINC components | (Stroud et al., 2018) |
|  | *Tmem43-p.S358L* (global CRISPR/Cas9 knock-in) | - Normal cardiac function |  |
|  | Conditional *Tmem43-p.S358L* (knock-in, *EIIα:Cre)* | - Structural cardiac abnormalities - Fibro-fatty infiltration - No inflammation - Increased NFκB/TGFβ signaling - Normal cardiac function | (Zheng et al., 2019) |
|  | *Myh6:**TMEM43-p.S358L, transgenic mouse* | - Severe biventricular cardiac dysfunction - Increased lethality - Fibro-fatty replacement of the myocardium - Cardiac conduction block | (Padron-Barthe et al., 2019) |
|  | *Myh6:TMEM43*-WT transgenic mouse, control | - No structural or functional defects |  |
| Sodium Voltage-Gated Channel α Subunit 5 (*SCN5A*) | Global *Scn5a* knock-out *Scn5a -/-*  *Scn5a -/+* | - Homozygous lethal - Severe defects in ventricular morphogenesis - Heterozygous hearts: impaired atrioventricular conduction, delayed intramyocardial conduction, increased ventricular refractoriness, and ventricular tachycardia with characteristics of re-entrant excitation | (Liu et al., 2006) |
|  | MHC-Snail transgenic mice [Myh6-Snai1]B10JCC  Repression of *Scn5a* after birth | - 90% reduction of *Scn5a* - Conduction defects - Significantly reduced voltage gated sodium current | (Hesse et al., 2007) |
|  | Human p.F1759A-NaV1.5  [Tg(Myh6*/tetO-SCN5A*p.F1759A)](http://www.informatics.jax.org/allele/MGI:5800483) | - Atrial and ventricular enlargement - Myofibrillar disarray, Fibrosis - Mitochondrial injury - Electrophysiological dysfunctions - Spontaneous and prolonged episodes of atrial fibrillation - Right atrial enlargement | (Wan et al., 2016) |
|  | *Scn5a-*p.S571E and p.S571A knock-in mice | - p.S571E/A: CaMKII-dependent phosphorylation abolished - S571 phosphorylation is necessary for maladaptive remodeling in response to pressure overload - S571 regulates susceptibility to arrhythmia events - S571 controls late sodium current at baseline | (Glynn et al., 2015) |
|  | Humanized *SCN5A*  p.D1275N knock-in  H/H - wild-type  DN/DN-homozygous | - Slow conduction, heart block, atrial fibrillation - ventricular tachycardia - DCM phenotype, with no significant fibrosis or myocyte disarray - reduced cardiac sodium current | (Watanabe et al., 2011) |
| Desmin (*DES*) | Global *Des* knock-out | - Biventricular cardiomyopathy - Cardiac fibrosis - Cardiac calcification and necrosis | (Li et al., 1996;Capetanaki et al., 1997;Psarras et al., 2012) |
|  | *Des*:HA-*Des*-p.L345P, transgenic mouse | - Left ventricular hypertrophy - No differences in fractional shortening - Increase of mitochondrial Ca^2+^ | (Kostareva et al., 2008) |
|  | *Des-*p.R349P (knock-in) | - Heart failure in heterozygous mice after transverse aortic constriction - Myocardial fibrosis - Transverse aortic constriction induce arrhythmia - Reduced activity of respiratory chain | (Stockigt et al., 2020) |
| Titin (*TTN*) | Knock-in *Ttn* 2-bp insertion of corresponding human mutation (*TTN* c.43628insAT) | - Leads to an unstable titin protein - Homozygous: lethal at E9.5, defects in sarcomere formation - Heterozygous: stress-induced cardiomyopathy | (Gramlich et al., 2009) |
| Phospholamban (*PLN*) | *Myh6:* *PLN-*p.R14del  (transgenic mouse) | - Died between 2-16 weeks - Ventricular dilation - Myocyte disarray - Myocardial fibrosis - Super-inhibition of the calcium affinity for SERCA2a | (Haghighi et al., 2006) |
|  | *Myh6:PLN-*WT (control) | - No phenotype |  |
|  | *Myh6:* *PLN-*p.R14del on the background of *Pln-*knock-out | - Hyperdynamic contractility - Progression to cardiac hypertrophy - Mutant PLN did not co-localize with SERCA2a instead at the plasma membrane - Altered function of the Na/K ATPase (NKA) - R14 is required for insertion in the SR membrane in the absence of wild-type PLN | (Chu et al., 1998;Haghighi et al., 2012) |
|  | *Pln-*knock out | - Hyperdynamic contractility - Attenuated responses to beta-agonists - No cytoarchitectural abnormalities |  |
| Lamin A/C (*LMNA*) | Global *Lmna*-knock-out  *Lmna +/-*  (CCS:lacZ in the conduction system) | - Develop to term with no overt abnormalities, but postnatal growth is severely retarded with appearance of muscular dystrophy - Ultrastructural perturbations to the nuclear envelope - EDMD phenotype - Rapidly progressive dilated cardiomyopathy - Myocyte nuclei have marked alterations of shape and size with central displacement and fragmentation of heterochromatin - Defective force transmission resulting from disruption of lamin interactions with the muscle-specific desmin network and loss of cytoskeletal tension - Heterozygous mice: atrioventricular (AV) conduction defects and both atrial and ventricular arrhythmia - Aged mice with exhibited impaired contractility without structural changes | (Sullivan et al., 1999;Nikolova et al., 2004;Wolf et al., 2008) |
| αT-Catenin (*CTNNA3*) | Conditional *Ctnna3* knock-out (*Cre*-deleter, ∆exon 3) | - Viable and fertile - Progressive cardiomyopathy - Left ventricular dilation - Reduced ejection fraction and fractional shortening - Reduced expression of plakophilin-2 and connexin-43 | (Li et al., 2012) |
| LIM Domain-Binding Protein-3 (*LDB3*) | Conditional cardiac-specific *Ldb3* knock-out (*Mlv2:Cre,* ∆exon 1) | - Biventricular dilation - Decreased cardiac function - Disrupted sarcomere ultrastructure - Increased mortality - Altered ERK / Stat3 signaling | (Zheng et al., 2009) |
|  | Conditional inducible cardiac-specific *Ldb3* knock-out (*Myh6:MerCreMer,* ∆exon 1) |  |  |
|  | *Myh6*:*LDB3*-p.S196L-His_6_-V5, transgenic mouse | - Biventricular dilation - Ventricular arrhythmia - Conduction abnormalities - Electrophysiological abnormalities - Mild cardiac fibrosis - Altered sarcomeric structure | (Li et al., 2010) |
| N-Cadherin (*CDH2*) | *Myh6*:*CDH2* (chicken) (transgenic mouse) | - Biventricular cardiomyopathy - Increase ANF expression - Decreased Connexin-43 expression | (Ferreira-Cornwell et al., 2002) |
|  | Conditional, inducible, cardiac specific *Cdh2* knock-out *(tamoxifen-inducible Cre)* | - Dilated cardiomyopathy - Premature death two months after induction - Sarcomere abnormalities - Ventricular arrhythmia - Conduction abnormalities - Decrease expression of connexion-43/-40 | (Kostetskii et al., 2005;Li et al., 2005) |
| RNA-Binding Motif Protein-20 (*RBM20*) | *Rattus norvegicus*  Homozygous, spontaneous 95 kb deletion affecting *rbm20* (∆exon 2-14) | - Titin (*ttn*) splice defect - Adenoviral mediated *rbm20* transduction rescues titin splicing in *rbm20* deficient cardiomyocytes - Sudden cardiac death (heterozygous & homozygous) - Left ventricular dilation (heterozygous & homozygous) - Cardiac fibrosis (heterozygous & homozygous) - Splicing of 31 cardiac genes is *rbm20* dependent (human & rat) | (Guo et al., 2012) |
|  | *Rbm20*-p.S637A (knock-in) | - *Ttn* splicing defects (heterozygous and homozygous) - No functional characterization | (Murayama et al., 2018) |
|  | Conditional *Rbm20* knock-out (*CMV:Cre*, ∆exon 4-5) | - Lack of titin circRNAs - Early-onset cardiomyopathy - Increased ANP / BNP expression (homozygous) - Decreased fractional shortening and ejection function (heterozygous and homozygous) - Cardiac fibrosis (homozygous) - Electrophysiological abnormalities - Spontaneous Ca^2+^ release (heterozygous and homozygous) - Disturbed Ca^2+^ handling - Splicing defects in *Ttn*, *Camk2d* and *Ryr2* | (Khan et al., 2016;van den Hoogenhof et al., 2018) |
| Tumor protein 63 (*TP63*) | Global *Tp63* knock-out  *p63^Brdm1^* | - No cardiac phenotype - Born alive but have striking developmental defects - Limbs are absent or truncated - Skin lacks stratification and does not express differentiation markers | (Mills et al., 1999) |
| LEM Domain-Containing Protein-2 (*LEMD2*) | Global *Lemd2* knock-out | - Embryonically lethal at E11.5 - Most tissues reduced in size - Activation of MAPK signaling - Embryonic hearts: thin myocardium, underdeveloped trabeculae | (Tapia et al., 2015) |
| Integrin Linked Kinase (*ILK*) | Conditional, muscle specific *Ilk* knock-out (*Mck:Cre;* loss of exon 5-12) | - Increase mortality - Ventricular dilation - Ventricular arrhythmia - Prolonged action potentials - Left-ventricular fibrosis - Reduced Akt phosphorylation - Connexin 43 down regulation | (White et al., 2006;Quang et al., 2015) |
| Filamin-C (*FLNC*) | Deletion of *Flnc* ∆exon 41-48 (homozygous) | - Mice die shortly after birth due to respiratory failure - Muscle fiber abnormalities - No functional or structural cardiac investigations | (Dalkilic et al., 2006) |
|  | Global *Flnc* knock-out | - Embryonic lethal | (Zhou et al., 2020) |
|  | Cardiac specific *Flnc* knock-out (*Tnnt2*:*Cre*) | - Embryonic lethal |  |
|  | Inducible, cardiac specific *Flnc* knock-out (*Myh6*:MerCreMer, ∆ exon 9-13) | - Cardiac dysfunction - Increased mortality - Cardiac fibrosis - Increased expression of costameric and intercalated disc proteins |  |
| Sorbin and SH3 Domain-Containing Protein-2 (*SORBS2*) | Knock-out (∆exon 8) of *Sorbs2* | - Dilation of the right ventricle - Right ventricular dysfunction - Arrhythmia - Premature cardiac death | (Ding et al., 2019) |

**Table 2.** Overview about zebrafish models for ACM.

| Protein (Gene) | Zebrafish Model | Major Finding | Reference |
| --- | --- | --- | --- |
| Desmocollin-2 (*DSC2*) | Knock-down of *dsc2l* (morpholinos) | - Cardiac dysfunction (fractional shortening) - Cardiac edema - Altered structure of the desmosomes | (Heuser et al., 2006) |
| Plakoglobin (*JUP*) | Knock-down of *jupa* (morpholinos) | - Decreased heart size - Decreased heart rate - Cardiac edema - Reflux of blood - Altered WNT signaling | (Martin et al., 2009) |
|  | *Gal4/UAS:JUP-*c.2057del2 and  *Gal4/UAS:JUP-*WT | - Premature death - Cardiac dilation - Cardiac dysfunction - Electrophysiological abnormalities - Reversal of electrophysiological abnormalities by GSK3β inhibition | (Asimaki et al., 2014) |
| Plakophilin-2 (*PKP2*) | Knock-down of *pkp2* (morpholinos) | - Decreased heart rate - Cardiac edema - Incomplete looping of the heart - Altered structure of the desmosomes | (Moriarty et al., 2012) |
| Desmoplakin (*DSP*) | Knock-down of *dspa* and *dspb* (morpholinos) | - Altered structure of the desmosomes - Decreased heart rate - Altered Wnt/β-catenin, TGFβ/Smad and Hippo/YAP-TAZ signaling | (Giuliodori et al., 2018) |
| Integrin Linked Kinase (*ILK*) | *cmlc2*:*ILK*-p.H33N-GFP; *cmlc2*:*ILK*-p.H77Y-GFP; *cmlc2*:*ILK*-WT-GFP (control), (transgenic zebrafish). | - Cardiac dysfunction (fractional shortening) - Premature death | (Brodehl et al., 2019) |
|  | Spontaneous missense mutation *ilk-*p.L308P^hom^ induced by ethylnitros-urea (ENU) | - Cardiac edema - Decreased fractional shortening - Mutation p.L308P disrupts binding to Z-disc protein parvin - Decreased kinase activity | (Bendig et al., 2006;Pott et al., 2018) |
| Sodium Voltage-Gated Channel α Subunit 5 (*SCN5A*) | *cmlc2*:*SCN5A p.D1275N* | - Bradycardia - Conduction-system abnormalities and premature death - No impaired cardiac function | (Huttner et al., 2013) |
|  | Knock-down of *scn5Laa* and *scn5Lab* via morpholinos | - Compromises both early chamber formation and normal patterned growth of the ventricle | (Chopra et al., 2010) |
| Lamin A/C (*LMNA*) | Knock-down of *lmna* via morpholinos | - Cardiomyopathic phenotype with significant bradycardia | (Vogel et al., 2009) |
|  | *lamin A* (zlamin A-Δ8) | - Embryonic senescence and S-phase accumulation/arrest - Abnormal muscle and lipodystrophic phenotypes - Heart not investigated | (Koshimizu et al., 2011) |
| Titin (*TTN*) | *ttna ^n/n^* and *ttna^c/c^* | - Homozygous: severe deficits in cardiac contractility, severe sarcomeric disarray - ~75–80% of mutant transcript being degraded - *ttna ^c/c^*: severe skeletal muscle disease; *ttna*^n/n^ normal skeletal muscle - Difference explained by an internal promoter producing the Cronos protein | (Zou et al., 2015) |
|  | *ttna*tv/+ | - Spontaneous development of DCM at 6 months of age - Hypercontractile response when challenged to hemodynamic stress | (Huttner et al., 2018) |
|  | Panel of *ttn* truncation mutants | - One null mutant *ttn^xu071^* and six hypomorph *ttn* mutants - *ttn^xu071^*: guiding the assembly of nascent myofibrils from premyofibrils - Hypomorph *ttn* mutants: susceptible to biomechanical stress or degenerated during development | (Shih et al., 2016) |
| Tumor protein 63 (*TP63*) | *tp63* knock-out | - Similar phenotype as in knock-out mice - Pioneer and repressive functions of p63 during   embryonic ectoderm specification | (Santos-Pereira et al., 2019) |

**References**

Asimaki, A., Kapoor, S., Plovie, E., Karin Arndt, A., Adams, E., Liu, Z., James, C.A., Judge, D.P., Calkins, H., Churko, J., Wu, J.C., Macrae, C.A., Kleber, A.G., and Saffitz, J.E. (2014). Identification of a new modulator of the intercalated disc in a zebrafish model of arrhythmogenic cardiomyopathy. *Sci Transl Med* 6**,** 240ra274.

Bendig, G., Grimmler, M., Huttner, I.G., Wessels, G., Dahme, T., Just, S., Trano, N., Katus, H.A., Fishman, M.C., and Rottbauer, W. (2006). Integrin-linked kinase, a novel component of the cardiac mechanical stretch sensor, controls contractility in the zebrafish heart. *Genes Dev* 20**,** 2361-2372.

Bierkamp, C., Mclaughlin, K.J., Schwarz, H., Huber, O., and Kemler, R. (1996). Embryonic heart and skin defects in mice lacking plakoglobin. *Dev Biol* 180**,** 780-785.

Brodehl, A., Belke, D.D., Garnett, L., Martens, K., Abdelfatah, N., Rodriguez, M., Diao, C., Chen, Y.X., Gordon, P.M., Nygren, A., and Gerull, B. (2017). Transgenic mice overexpressing desmocollin-2 (DSC2) develop cardiomyopathy associated with myocardial inflammation and fibrotic remodeling. *PLoS One* 12**,** e0174019.

Brodehl, A., Rezazadeh, S., Williams, T., Munsie, N.M., Liedtke, D., Oh, T., Ferrier, R., Shen, Y., Jones, S.J.M., Stiegler, A.L., Boggon, T.J., Duff, H.J., Friedman, J.M., Gibson, W.T., Consortium, F.C., Childs, S.J., and Gerull, B. (2019). Mutations in ILK, encoding integrin-linked kinase, are associated with arrhythmogenic cardiomyopathy. *Transl Res* 208**,** 15-29.

Bround, M.J., Asghari, P., Wambolt, R.B., Bohunek, L., Smits, C., Philit, M., Kieffer, T.J., Lakatta, E.G., Boheler, K.R., Moore, E.D., Allard, M.F., and Johnson, J.D. (2012). Cardiac ryanodine receptors control heart rate and rhythmicity in adult mice. *Cardiovasc Res* 96**,** 372-380.

Buck, V.U., Hodecker, M., Eisner, S., Leube, R.E., Krusche, C.A., and Classen-Linke, I. (2018). Ultrastructural changes in endometrial desmosomes of desmoglein 2 mutant mice. *Cell Tissue Res* 374**,** 317-327.

Calore, M., Lorenzon, A., Vitiello, L., Poloni, G., Khan, M.a.F., Beffagna, G., Dazzo, E., Sacchetto, C., Polishchuk, R., Sabatelli, P., Doliana, R., Carnevale, D., Lembo, G., Bonaldo, P., De Windt, L., Braghetta, P., and Rampazzo, A. (2019). A novel murine model for arrhythmogenic cardiomyopathy points to a pathogenic role of Wnt signalling and miRNA dysregulation. *Cardiovasc Res* 115**,** 739-751.

Capetanaki, Y., Milner, D.J., and Weitzer, G. (1997). Desmin in muscle formation and maintenance: knockouts and consequences. *Cell Struct Funct* 22**,** 103-116.

Cerrone, M., Montnach, J., Lin, X., Zhao, Y.T., Zhang, M., Agullo-Pascual, E., Leo-Macias, A., Alvarado, F.J., Dolgalev, I., Karathanos, T.V., Malkani, K., Van Opbergen, C.J.M., Van Bavel, J.J.A., Yang, H.Q., Vasquez, C., Tester, D., Fowler, S., Liang, F., Rothenberg, E., Heguy, A., Morley, G.E., Coetzee, W.A., Trayanova, N.A., Ackerman, M.J., Van Veen, T.a.B., Valdivia, H.H., and Delmar, M. (2017). Plakophilin-2 is required for transcription of genes that control calcium cycling and cardiac rhythm. *Nat Commun* 8**,** 106.

Cerrone, M., Noorman, M., Lin, X., Chkourko, H., Liang, F.X., Van Der Nagel, R., Hund, T., Birchmeier, W., Mohler, P., Van Veen, T.A., Van Rijen, H.V., and Delmar, M. (2012). Sodium current deficit and arrhythmogenesis in a murine model of plakophilin-2 haploinsufficiency. *Cardiovasc Res* 95**,** 460-468.

Cheedipudi, S.M., Hu, J., Fan, S., Yuan, P., Karmouch, J., Czernuszewicz, G., Robertson, M.J., Coarfa, C., Hong, K., Yao, Y., Moore, H.C., Wehrens, X., Gurha, P., and Marian, A.J. (2019). Exercise Restores Dysregulated Gene Expression in a Mouse Model of Arrhythmogenic Cardiomyopathy. *Cardiovasc Res*.

Chelko, S.P., Asimaki, A., Andersen, P., Bedja, D., Amat-Alarcon, N., Demazumder, D., Jasti, R., Macrae, C.A., Leber, R., Kleber, A.G., Saffitz, J.E., and Judge, D.P. (2016). Central role for GSK3beta in the pathogenesis of arrhythmogenic cardiomyopathy. *JCI Insight* 1.

Chopra, S.S., Stroud, D.M., Watanabe, H., Bennett, J.S., Burns, C.G., Wells, K.S., Yang, T., Zhong, T.P., and Roden, D.M. (2010). Voltage-gated sodium channels are required for heart development in zebrafish. *Circ Res* 106**,** 1342-1350.

Chu, G., Ferguson, D.G., Edes, I., Kiss, E., Sato, Y., and Kranias, E.G. (1998). Phospholamban ablation and compensatory responses in the mammalian heart. *Ann N Y Acad Sci* 853**,** 49-62.

Cruz, F.M., Sanz-Rosa, D., Roche-Molina, M., Garcia-Prieto, J., Garcia-Ruiz, J.M., Pizarro, G., Jimenez-Borreguero, L.J., Torres, M., Bernad, A., Ruiz-Cabello, J., Fuster, V., Ibanez, B., and Bernal, J.A. (2015). Exercise triggers ARVC phenotype in mice expressing a disease-causing mutated version of human plakophilin-2. *J Am Coll Cardiol* 65**,** 1438-1450.

Dalkilic, I., Schienda, J., Thompson, T.G., and Kunkel, L.M. (2006). Loss of FilaminC (FLNc) results in severe defects in myogenesis and myotube structure. *Mol Cell Biol* 26**,** 6522-6534.

Ding, Y., Yang, J., Chen, P., Lu, T., Jiao, K., Tester, D., Jiang, K., Ackerman, M.J., Li, Y., and Wang, D.W. (2019). SORBS2 is a susceptibility gene to arrhythmogenic right ventricular cardiomyopathy. *bioRxiv***,** 725077.

Eshkind, L., Tian, Q., Schmidt, A., Franke, W.W., Windoffer, R., and Leube, R.E. (2002). Loss of desmoglein 2 suggests essential functions for early embryonic development and proliferation of embryonal stem cells. *Eur J Cell Biol* 81**,** 592-598.

Fabritz, L., Hoogendijk, M.G., Scicluna, B.P., Van Amersfoorth, S.C., Fortmueller, L., Wolf, S., Laakmann, S., Kreienkamp, N., Piccini, I., Breithardt, G., Noppinger, P.R., Witt, H., Ebnet, K., Wichter, T., Levkau, B., Franke, W.W., Pieperhoff, S., De Bakker, J.M., Coronel, R., and Kirchhof, P. (2011). Load-reducing therapy prevents development of arrhythmogenic right ventricular cardiomyopathy in plakoglobin-deficient mice. *J Am Coll Cardiol* 57**,** 740-750.

Ferreira-Cornwell, M.C., Luo, Y., Narula, N., Lenox, J.M., Lieberman, M., and Radice, G.L. (2002). Remodeling the intercalated disc leads to cardiomyopathy in mice misexpressing cadherins in the heart. *J Cell Sci* 115**,** 1623-1634.

Gallicano, G.I., Kouklis, P., Bauer, C., Yin, M., Vasioukhin, V., Degenstein, L., and Fuchs, E. (1998). Desmoplakin is required early in development for assembly of desmosomes and cytoskeletal linkage. *J Cell Biol* 143**,** 2009-2022.

Garcia-Gras, E., Lombardi, R., Giocondo, M.J., Willerson, J.T., Schneider, M.D., Khoury, D.S., and Marian, A.J. (2006). Suppression of canonical Wnt/beta-catenin signaling by nuclear plakoglobin recapitulates phenotype of arrhythmogenic right ventricular cardiomyopathy. *J Clin Invest* 116**,** 2012-2021.

Giuliodori, A., Beffagna, G., Marchetto, G., Fornetto, C., Vanzi, F., Toppo, S., Facchinello, N., Santimaria, M., Vettori, A., Rizzo, S., Della Barbera, M., Pilichou, K., Argenton, F., Thiene, G., Tiso, N., and Basso, C. (2018). Loss of cardiac Wnt/beta-catenin signalling in desmoplakin-deficient AC8 zebrafish models is rescuable by genetic and pharmacological intervention. *Cardiovasc Res* 114**,** 1082-1097.

Glynn, P., Musa, H., Wu, X., Unudurthi, S.D., Little, S., Qian, L., Wright, P.J., Radwanski, P.B., Gyorke, S., Mohler, P.J., and Hund, T.J. (2015). Voltage-Gated Sodium Channel Phosphorylation at Ser571 Regulates Late Current, Arrhythmia, and Cardiac Function In Vivo. *Circulation* 132**,** 567-577.

Gomes, J., Finlay, M., Ahmed, A.K., Ciaccio, E.J., Asimaki, A., Saffitz, J.E., Quarta, G., Nobles, M., Syrris, P., Chaubey, S., Mckenna, W.J., Tinker, A., and Lambiase, P.D. (2012). Electrophysiological abnormalities precede overt structural changes in arrhythmogenic right ventricular cardiomyopathy due to mutations in desmoplakin-A combined murine and human study. *Eur Heart J* 33**,** 1942-1953.

Gramlich, M., Michely, B., Krohne, C., Heuser, A., Erdmann, B., Klaassen, S., Hudson, B., Magarin, M., Kirchner, F., Todiras, M., Granzier, H., Labeit, S., Thierfelder, L., and Gerull, B. (2009). Stress-induced dilated cardiomyopathy in a knock-in mouse model mimicking human titin-based disease. *J Mol Cell Cardiol* 47**,** 352-358.

Grossmann, K.S., Grund, C., Huelsken, J., Behrend, M., Erdmann, B., Franke, W.W., and Birchmeier, W. (2004). Requirement of plakophilin 2 for heart morphogenesis and cardiac junction formation. *J Cell Biol* 167**,** 149-160.

Guo, W., Schafer, S., Greaser, M.L., Radke, M.H., Liss, M., Govindarajan, T., Maatz, H., Schulz, H., Li, S., Parrish, A.M., Dauksaite, V., Vakeel, P., Klaassen, S., Gerull, B., Thierfelder, L., Regitz-Zagrosek, V., Hacker, T.A., Saupe, K.W., Dec, G.W., Ellinor, P.T., Macrae, C.A., Spallek, B., Fischer, R., Perrot, A., Ozcelik, C., Saar, K., Hubner, N., and Gotthardt, M. (2012). RBM20, a gene for hereditary cardiomyopathy, regulates titin splicing. *Nat Med* 18**,** 766-773.

Haghighi, K., Kolokathis, F., Gramolini, A.O., Waggoner, J.R., Pater, L., Lynch, R.A., Fan, G.C., Tsiapras, D., Parekh, R.R., Dorn, G.W., 2nd, Maclennan, D.H., Kremastinos, D.T., and Kranias, E.G. (2006). A mutation in the human phospholamban gene, deleting arginine 14, results in lethal, hereditary cardiomyopathy. *Proc Natl Acad Sci U S A* 103**,** 1388-1393.

Haghighi, K., Pritchard, T., Bossuyt, J., Waggoner, J.R., Yuan, Q., Fan, G.C., Osinska, H., Anjak, A., Rubinstein, J., Robbins, J., Bers, D.M., and Kranias, E.G. (2012). The human phospholamban Arg14-deletion mutant localizes to plasma membrane and interacts with the Na/K-ATPase. *J Mol Cell Cardiol* 52**,** 773-782.

Hamada, Y., Yamamoto, T., Nakamura, Y., Sufu-Shimizu, Y., Nanno, T., Fukuda, M., Ono, M., Oda, T., Okuda, S., Ueyama, T., Kobayashi, S., and Yano, M. (2020). G790del mutation in DSC2 alone is insufficient to develop the pathogenesis of ARVC in a mouse model. *Biochem Biophys Rep* 21**,** 100711.

Herbert Pratt, C., Potter, C.S., Fairfield, H., Reinholdt, L.G., Bergstrom, D.E., Harris, B.S., Greenstein, I., Dadras, S.S., Liang, B.T., Schofield, P.N., and Sundberg, J.P. (2015). Dsp rul: a spontaneous mouse mutation in desmoplakin as a model of Carvajal-Huerta syndrome. *Exp Mol Pathol* 98**,** 164-172.

Hesse, M., Kondo, C.S., Clark, R.B., Su, L., Allen, F.L., Geary-Joo, C.T., Kunnathu, S., Severson, D.L., Nygren, A., Giles, W.R., and Cross, J.C. (2007). Dilated cardiomyopathy is associated with reduced expression of the cardiac sodium channel Scn5a. *Cardiovasc Res* 75**,** 498-509.

Heuser, A., Plovie, E.R., Ellinor, P.T., Grossmann, K.S., Shin, J.T., Wichter, T., Basson, C.T., Lerman, B.B., Sasse-Klaassen, S., Thierfelder, L., Macrae, C.A., and Gerull, B. (2006). Mutant desmocollin-2 causes arrhythmogenic right ventricular cardiomyopathy. *Am J Hum Genet* 79**,** 1081-1088.

Huttner, I.G., Trivedi, G., Jacoby, A., Mann, S.A., Vandenberg, J.I., and Fatkin, D. (2013). A transgenic zebrafish model of a human cardiac sodium channel mutation exhibits bradycardia, conduction-system abnormalities and early death. *J Mol Cell Cardiol* 61**,** 123-132.

Huttner, I.G., Wang, L.W., Santiago, C.F., Horvat, C., Johnson, R., Cheng, D., Von Frieling-Salewsky, M., Hillcoat, K., Bemand, T.J., Trivedi, G., Braet, F., Hesselson, D., Alford, K., Hayward, C.S., Seidman, J.G., Seidman, C.E., Feneley, M.P., Linke, W.A., and Fatkin, D. (2018). A-Band Titin Truncation in Zebrafish Causes Dilated Cardiomyopathy and Hemodynamic Stress Intolerance. *Circ Genom Precis Med* 11**,** e002135.

Kaartinen, V., Voncken, J.W., Shuler, C., Warburton, D., Bu, D., Heisterkamp, N., and Groffen, J. (1995). Abnormal lung development and cleft palate in mice lacking TGF-beta 3 indicates defects of epithelial-mesenchymal interaction. *Nat Genet* 11**,** 415-421.

Kannankeril, P.J., Mitchell, B.M., Goonasekera, S.A., Chelu, M.G., Zhang, W., Sood, S., Kearney, D.L., Danila, C.I., De Biasi, M., Wehrens, X.H., Pautler, R.G., Roden, D.M., Taffet, G.E., Dirksen, R.T., Anderson, M.E., and Hamilton, S.L. (2006). Mice with the R176Q cardiac ryanodine receptor mutation exhibit catecholamine-induced ventricular tachycardia and cardiomyopathy. *Proc Natl Acad Sci U S A* 103**,** 12179-12184.

Kant, S., Holthofer, B., Magin, T.M., Krusche, C.A., and Leube, R.E. (2015). Desmoglein 2-Dependent Arrhythmogenic Cardiomyopathy Is Caused by a Loss of Adhesive Function. *Circ Cardiovasc Genet* 8**,** 553-563.

Kant, S., Krull, P., Eisner, S., Leube, R.E., and Krusche, C.A. (2012). Histological and ultrastructural abnormalities in murine desmoglein 2-mutant hearts. *Cell Tissue Res* 348**,** 249-259.

Khan, M.A., Reckman, Y.J., Aufiero, S., Van Den Hoogenhof, M.M., Van Der Made, I., Beqqali, A., Koolbergen, D.R., Rasmussen, T.B., Van Der Velden, J., Creemers, E.E., and Pinto, Y.M. (2016). RBM20 Regulates Circular RNA Production From the Titin Gene. *Circ Res* 119**,** 996-1003.

Kirchhof, P., Fabritz, L., Zwiener, M., Witt, H., Schafers, M., Zellerhoff, S., Paul, M., Athai, T., Hiller, K.H., Baba, H.A., Breithardt, G., Ruiz, P., Wichter, T., and Levkau, B. (2006). Age- and training-dependent development of arrhythmogenic right ventricular cardiomyopathy in heterozygous plakoglobin-deficient mice. *Circulation* 114**,** 1799-1806.

Koshimizu, E., Imamura, S., Qi, J., Toure, J., Valdez, D.M., Jr., Carr, C.E., Hanai, J., and Kishi, S. (2011). Embryonic senescence and laminopathies in a progeroid zebrafish model. *PLoS One* 6**,** e17688.

Kostareva, A., Sjoberg, G., Bruton, J., Zhang, S.J., Balogh, J., Gudkova, A., Hedberg, B., Edstrom, L., Westerblad, H., and Sejersen, T. (2008). Mice expressing L345P mutant desmin exhibit morphological and functional changes of skeletal and cardiac mitochondria. *J Muscle Res Cell Motil* 29**,** 25-36.

Kostetskii, I., Li, J., Xiong, Y., Zhou, R., Ferrari, V.A., Patel, V.V., Molkentin, J.D., and Radice, G.L. (2005). Induced deletion of the N-cadherin gene in the heart leads to dissolution of the intercalated disc structure. *Circ Res* 96**,** 346-354.

Krusche, C.A., Holthofer, B., Hofe, V., Van De Sandt, A.M., Eshkind, L., Bockamp, E., Merx, M.W., Kant, S., Windoffer, R., and Leube, R.E. (2011). Desmoglein 2 mutant mice develop cardiac fibrosis and dilation. *Basic Res Cardiol* 106**,** 617-633.

Leo-Macias, A., Liang, F.X., and Delmar, M. (2015). Ultrastructure of the intercellular space in adult murine ventricle revealed by quantitative tomographic electron microscopy. *Cardiovasc Res* 107**,** 442-452.

Li, D., Liu, Y., Maruyama, M., Zhu, W., Chen, H., Zhang, W., Reuter, S., Lin, S.F., Haneline, L.S., Field, L.J., Chen, P.S., and Shou, W. (2011a). Restrictive loss of plakoglobin in cardiomyocytes leads to arrhythmogenic cardiomyopathy. *Hum Mol Genet* 20**,** 4582-4596.

Li, J., Goossens, S., Van Hengel, J., Gao, E., Cheng, L., Tyberghein, K., Shang, X., De Rycke, R., Van Roy, F., and Radice, G.L. (2012). Loss of alphaT-catenin alters the hybrid adhering junctions in the heart and leads to dilated cardiomyopathy and ventricular arrhythmia following acute ischemia. *J Cell Sci* 125**,** 1058-1067.

Li, J., Patel, V.V., Kostetskii, I., Xiong, Y., Chu, A.F., Jacobson, J.T., Yu, C., Morley, G.E., Molkentin, J.D., and Radice, G.L. (2005). Cardiac-specific loss of N-cadherin leads to alteration in connexins with conduction slowing and arrhythmogenesis. *Circ Res* 97**,** 474-481.

Li, J., Swope, D., Raess, N., Cheng, L., Muller, E.J., and Radice, G.L. (2011b). Cardiac tissue-restricted deletion of plakoglobin results in progressive cardiomyopathy and activation of {beta}-catenin signaling. *Mol Cell Biol* 31**,** 1134-1144.

Li, Z., Ai, T., Samani, K., Xi, Y., Tzeng, H.P., Xie, M., Wu, S., Ge, S., Taylor, M.D., Dong, J.W., Cheng, J., Ackerman, M.J., Kimura, A., Sinagra, G., Brunelli, L., Faulkner, G., and Vatta, M. (2010). A ZASP missense mutation, S196L, leads to cytoskeletal and electrical abnormalities in a mouse model of cardiomyopathy. *Circ Arrhythm Electrophysiol* 3**,** 646-656.

Li, Z., Colucci-Guyon, E., Pincon-Raymond, M., Mericskay, M., Pournin, S., Paulin, D., and Babinet, C. (1996). Cardiovascular lesions and skeletal myopathy in mice lacking desmin. *Dev Biol* 175**,** 362-366.

Liu, K., Hipkens, S., Yang, T., Abraham, R., Zhang, W., Chopra, N., Knollmann, B., Magnuson, M.A., and Roden, D.M. (2006). Recombinase-mediated cassette exchange to rapidly and efficiently generate mice with human cardiac sodium channels. *Genesis* 44**,** 556-564.

Lombardi, R., Da Graca Cabreira-Hansen, M., Bell, A., Fromm, R.R., Willerson, J.T., and Marian, A.J. (2011). Nuclear plakoglobin is essential for differentiation of cardiac progenitor cells to adipocytes in arrhythmogenic right ventricular cardiomyopathy. *Circ Res* 109**,** 1342-1353.

Lyon, R.C., Mezzano, V., Wright, A.T., Pfeiffer, E., Chuang, J., Banares, K., Castaneda, A., Ouyang, K., Cui, L., Contu, R., Gu, Y., Evans, S.M., Omens, J.H., Peterson, K.L., Mcculloch, A.D., and Sheikh, F. (2014). Connexin defects underlie arrhythmogenic right ventricular cardiomyopathy in a novel mouse model. *Hum Mol Genet* 23**,** 1134-1150.

Martin, E.D., Moriarty, M.A., Byrnes, L., and Grealy, M. (2009). Plakoglobin has both structural and signalling roles in zebrafish development. *Dev Biol* 327**,** 83-96.

Mezzano, V., Liang, Y., Wright, A.T., Lyon, R.C., Pfeiffer, E., Song, M.Y., Gu, Y., Dalton, N.D., Scheinman, M., Peterson, K.L., Evans, S.M., Fowler, S., Cerrone, M., Mcculloch, A.D., and Sheikh, F. (2016). Desmosomal junctions are necessary for adult sinus node function. *Cardiovasc Res* 111**,** 274-286.

Mills, A.A., Zheng, B., Wang, X.J., Vogel, H., Roop, D.R., and Bradley, A. (1999). p63 is a p53 homologue required for limb and epidermal morphogenesis. *Nature* 398**,** 708-713.

Moncayo-Arlandi, J., Guasch, E., Sanz-De La Garza, M., Casado, M., Garcia, N.A., Mont, L., Sitges, M., Knoll, R., Buyandelger, B., Campuzano, O., Diez-Juan, A., and Brugada, R. (2016). Molecular disturbance underlies to arrhythmogenic cardiomyopathy induced by transgene content, age and exercise in a truncated PKP2 mouse model. *Hum Mol Genet* 25**,** 3676-3688.

Moriarty, M.A., Ryan, R., Lalor, P., Dockery, P., Byrnes, L., and Grealy, M. (2012). Loss of plakophilin 2 disrupts heart development in zebrafish. *Int J Dev Biol* 56**,** 711-718.

Murayama, R., Kimura-Asami, M., Togo-Ohno, M., Yamasaki-Kato, Y., Naruse, T.K., Yamamoto, T., Hayashi, T., Ai, T., Spoonamore, K.G., Kovacs, R.J., Vatta, M., Iizuka, M., Saito, M., Wani, S., Hiraoka, Y., Kimura, A., and Kuroyanagi, H. (2018). Phosphorylation of the RSRSP stretch is critical for splicing regulation by RNA-Binding Motif Protein 20 (RBM20) through nuclear localization. *Sci Rep* 8**,** 8970.

Nikolova, V., Leimena, C., Mcmahon, A.C., Tan, J.C., Chandar, S., Jogia, D., Kesteven, S.H., Michalicek, J., Otway, R., Verheyen, F., Rainer, S., Stewart, C.L., Martin, D., Feneley, M.P., and Fatkin, D. (2004). Defects in nuclear structure and function promote dilated cardiomyopathy in lamin A/C-deficient mice. *J Clin Invest* 113**,** 357-369.

Padron-Barthe, L., Villalba-Orero, M., Gomez-Salinero, J.M., Dominguez, F., Roman, M., Larrasa-Alonso, J., Ortiz-Sanchez, P., Martinez, F., Lopez-Olaneta, M., Bonzon-Kulichenko, E., Vazquez, J., Marti-Gomez, C., Santiago, D.J., Prados, B., Giovinazzo, G., Gomez-Gaviro, M.V., Priori, S., Garcia-Pavia, P., and Lara-Pezzi, E. (2019). Severe Cardiac Dysfunction and Death Caused by Arrhythmogenic Right Ventricular Cardiomyopathy Type 5 Are Improved by Inhibition of Glycogen Synthase Kinase-3beta. *Circulation* 140**,** 1188-1204.

Pilichou, K., Remme, C.A., Basso, C., Campian, M.E., Rizzo, S., Barnett, P., Scicluna, B.P., Bauce, B., Van Den Hoff, M.J., De Bakker, J.M., Tan, H.L., Valente, M., Nava, A., Wilde, A.A., Moorman, A.F., Thiene, G., and Bezzina, C.R. (2009). Myocyte necrosis underlies progressive myocardial dystrophy in mouse dsg2-related arrhythmogenic right ventricular cardiomyopathy. *J Exp Med* 206**,** 1787-1802.

Pott, A., Shahid, M., Kohler, D., Pylatiuk, C., Weinmann, K., Just, S., and Rottbauer, W. (2018). Therapeutic Chemical Screen Identifies Phosphatase Inhibitors to Reconstitute PKB Phosphorylation and Cardiac Contractility in ILK-Deficient Zebrafish. *Biomolecules* 8.

Proetzel, G., Pawlowski, S.A., Wiles, M.V., Yin, M., Boivin, G.P., Howles, P.N., Ding, J., Ferguson, M.W., and Doetschman, T. (1995). Transforming growth factor-beta 3 is required for secondary palate fusion. *Nat Genet* 11**,** 409-414.

Psarras, S., Mavroidis, M., Sanoudou, D., Davos, C.H., Xanthou, G., Varela, A.E., Panoutsakopoulou, V., and Capetanaki, Y. (2012). Regulation of adverse remodelling by osteopontin in a genetic heart failure model. *Eur Heart J* 33**,** 1954-1963.

Quang, K.L., Maguy, A., Qi, X.Y., Naud, P., Xiong, F., Tadevosyan, A., Shi, Y.F., Chartier, D., Tardif, J.C., Dobrev, D., and Nattel, S. (2015). Loss of cardiomyocyte integrin-linked kinase produces an arrhythmogenic cardiomyopathy in mice. *Circ Arrhythm Electrophysiol* 8**,** 921-932.

Rizzo, S., Lodder, E.M., Verkerk, A.O., Wolswinkel, R., Beekman, L., Pilichou, K., Basso, C., Remme, C.A., Thiene, G., and Bezzina, C.R. (2012). Intercalated disc abnormalities, reduced Na(+) current density, and conduction slowing in desmoglein-2 mutant mice prior to cardiomyopathic changes. *Cardiovasc Res* 95**,** 409-418.

Ruiz, P., Brinkmann, V., Ledermann, B., Behrend, M., Grund, C., Thalhammer, C., Vogel, F., Birchmeier, C., Gunthert, U., Franke, W.W., and Birchmeier, W. (1996). Targeted mutation of plakoglobin in mice reveals essential functions of desmosomes in the embryonic heart. *J Cell Biol* 135**,** 215-225.

Santos-Pereira, J.M., Gallardo-Fuentes, L., Neto, A., Acemel, R.D., and Tena, J.J. (2019). Pioneer and repressive functions of p63 during zebrafish embryonic ectoderm specification. *Nat Commun* 10**,** 3049.

Shan, J., Xie, W., Betzenhauser, M., Reiken, S., Chen, B.X., Wronska, A., and Marks, A.R. (2012). Calcium leak through ryanodine receptors leads to atrial fibrillation in 3 mouse models of catecholaminergic polymorphic ventricular tachycardia. *Circ Res* 111**,** 708-717.

Shih, Y.H., Dvornikov, A.V., Zhu, P., Ma, X., Kim, M., Ding, Y., and Xu, X. (2016). Exon- and contraction-dependent functions of titin in sarcomere assembly. *Development* 143**,** 4713-4722.

Stockigt, F., Eichhorn, L., Beiert, T., Knappe, V., Radecke, T., Steinmetz, M., Nickenig, G., Peeva, V., Kudin, A.P., Kunz, W.S., Berwanger, C., Kamm, L., Schultheis, D., Schlotzer-Schrehardt, U., Clemen, C.S., Schroder, R., and Schrickel, J.W. (2020). Heart failure after pressure overload in autosomal-dominant desminopathies: Lessons from heterozygous DES-p.R349P knock-in mice. *PLoS One* 15**,** e0228913.

Stroud, M.J., Fang, X., Zhang, J., Guimaraes-Camboa, N., Veevers, J., Dalton, N.D., Gu, Y., Bradford, W.H., Peterson, K.L., Evans, S.M., Gerace, L., and Chen, J. (2018). Luma is not essential for murine cardiac development and function. *Cardiovasc Res* 114**,** 378-388.

Sullivan, T., Escalante-Alcalde, D., Bhatt, H., Anver, M., Bhat, N., Nagashima, K., Stewart, C.L., and Burke, B. (1999). Loss of A-type lamin expression compromises nuclear envelope integrity leading to muscular dystrophy. *J Cell Biol* 147**,** 913-920.

Takeshima, H., Komazaki, S., Hirose, K., Nishi, M., Noda, T., and Iino, M. (1998). Embryonic lethality and abnormal cardiac myocytes in mice lacking ryanodine receptor type 2. *EMBO J* 17**,** 3309-3316.

Tapia, O., Fong, L.G., Huber, M.D., Young, S.G., and Gerace, L. (2015). Nuclear envelope protein Lem2 is required for mouse development and regulates MAP and AKT kinases. *PLoS One* 10**,** e0116196.

Van Den Hoogenhof, M.M.G., Beqqali, A., Amin, A.S., Van Der Made, I., Aufiero, S., Khan, M.a.F., Schumacher, C.A., Jansweijer, J.A., Van Spaendonck-Zwarts, K.Y., Remme, C.A., Backs, J., Verkerk, A.O., Baartscheer, A., Pinto, Y.M., and Creemers, E.E. (2018). RBM20 Mutations Induce an Arrhythmogenic Dilated Cardiomyopathy Related to Disturbed Calcium Handling. *Circulation* 138**,** 1330-1342.

Vogel, B., Meder, B., Just, S., Laufer, C., Berger, I., Weber, S., Katus, H.A., and Rottbauer, W. (2009). In-vivo characterization of human dilated cardiomyopathy genes in zebrafish. *Biochem Biophys Res Commun* 390**,** 516-522.

Wan, E., Abrams, J., Weinberg, R.L., Katchman, A.N., Bayne, J., Zakharov, S.I., Yang, L., Morrow, J.P., Garan, H., and Marx, S.O. (2016). Aberrant sodium influx causes cardiomyopathy and atrial fibrillation in mice. *J Clin Invest* 126**,** 112-122.

Watanabe, H., Yang, T., Stroud, D.M., Lowe, J.S., Harris, L., Atack, T.C., Wang, D.W., Hipkens, S.B., Leake, B., Hall, L., Kupershmidt, S., Chopra, N., Magnuson, M.A., Tanabe, N., Knollmann, B.C., George, A.L., Jr., and Roden, D.M. (2011). Striking In vivo phenotype of a disease-associated human SCN5A mutation producing minimal changes in vitro. *Circulation* 124**,** 1001-1011.

White, D.E., Coutu, P., Shi, Y.F., Tardif, J.C., Nattel, S., St Arnaud, R., Dedhar, S., and Muller, W.J. (2006). Targeted ablation of ILK from the murine heart results in dilated cardiomyopathy and spontaneous heart failure. *Genes Dev* 20**,** 2355-2360.

Wolf, C.M., Wang, L., Alcalai, R., Pizard, A., Burgon, P.G., Ahmad, F., Sherwood, M., Branco, D.M., Wakimoto, H., Fishman, G.I., See, V., Stewart, C.L., Conner, D.A., Berul, C.I., Seidman, C.E., and Seidman, J.G. (2008). Lamin A/C haploinsufficiency causes dilated cardiomyopathy and apoptosis-triggered cardiac conduction system disease. *J Mol Cell Cardiol* 44**,** 293-303.

Yang, Z., Bowles, N.E., Scherer, S.E., Taylor, M.D., Kearney, D.L., Ge, S., Nadvoretskiy, V.V., Defreitas, G., Carabello, B., Brandon, L.I., Godsel, L.M., Green, K.J., Saffitz, J.E., Li, H., Danieli, G.A., Calkins, H., Marcus, F., and Towbin, J.A. (2006). Desmosomal dysfunction due to mutations in desmoplakin causes arrhythmogenic right ventricular dysplasia/cardiomyopathy. *Circ Res* 99**,** 646-655.

Zhang, Z., Stroud, M.J., Zhang, J., Fang, X., Ouyang, K., Kimura, K., Mu, Y., Dalton, N.D., Gu, Y., Bradford, W.H., Peterson, K.L., Cheng, H., Zhou, X., and Chen, J. (2015). Normalization of Naxos plakoglobin levels restores cardiac function in mice. *J Clin Invest* 125**,** 1708-1712.

Zheng, G., Jiang, C., Li, Y., Yang, D., Ma, Y., Zhang, B., Li, X., Zhang, P., Hu, X., Zhao, X., Du, J., and Lin, X. (2019). TMEM43-S358L mutation enhances NF-kappaB-TGFbeta signal cascade in arrhythmogenic right ventricular dysplasia/cardiomyopathy. *Protein Cell* 10**,** 104-119.

Zheng, M., Cheng, H., Li, X., Zhang, J., Cui, L., Ouyang, K., Han, L., Zhao, T., Gu, Y., Dalton, N.D., Bang, M.L., Peterson, K.L., and Chen, J. (2009). Cardiac-specific ablation of Cypher leads to a severe form of dilated cardiomyopathy with premature death. *Hum Mol Genet* 18**,** 701-713.

Zhou, Y., Chen, Z., Zhang, L., Zhu, M., Tan, C., Zhou, X., Evans, S.M., Fang, X., Feng, W., and Chen, J. (2020). Loss of Filamin C Is Catastrophic for Heart Function. *Circulation* 141**,** 869-871.

Zou, J., Tran, D., Baalbaki, M., Tang, L.F., Poon, A., Pelonero, A., Titus, E.W., Yuan, C., Shi, C., Patchava, S., Halper, E., Garg, J., Movsesyan, I., Yin, C., Wu, R., Wilsbacher, L.D., Liu, J., Hager, R.L., Coughlin, S.R., Jinek, M., Pullinger, C.R., Kane, J.P., Hart, D.O., Kwok, P.Y., and Deo, R.C. (2015). An internal promoter underlies the difference in disease severity between N- and C-terminal truncation mutations of Titin in zebrafish. *Elife* 4**,** e09406.
